# Comparison of a Rat Primary Cell-Based Blood-Brain Barrier Model With Epithelial and Brain Endothelial Cell Lines: Gene Expression and Drug Transport

**DOI:** 10.3389/fnmol.2018.00166

**Published:** 2018-05-22

**Authors:** Szilvia Veszelka, András Tóth, Fruzsina R. Walter, Andrea E. Tóth, Ilona Gróf, Mária Mészáros, Alexandra Bocsik, Éva Hellinger, Monika Vastag, Gábor Rákhely, Mária A. Deli

**Affiliations:** ^1^Biological Barriers Research Group, Institute of Biophysics, Biological Research Centre, Hungarian Academy of Sciences, Szeged, Hungary; ^2^Department of Biotechnology, Faculty of Science and Informatics, University of Szeged, Szeged, Hungary; ^3^Doctoral School in Biology, Faculty of Science and Informatics, University of Szeged, Szeged, Hungary; ^4^Doctoral School in Theoretical Medicine, Faculty of Medicine, University of Szeged, Szeged, Hungary; ^5^In Vitro Metabolism Research, Division of Pharmacology and Drug Safety, Gedeon Richter Plc., Budapest, Hungary

**Keywords:** blood-brain barrier, brain endothelial cells, Caco-2, MDCK, RBE4, hCMEC/D3, gene expression, CNS drug permeability

## Abstract

Cell culture-based blood-brain barrier (BBB) models are useful tools for screening of CNS drug candidates. Cell sources for BBB models include primary brain endothelial cells or immortalized brain endothelial cell lines. Despite their well-known differences, epithelial cell lines are also used as surrogate models for testing neuropharmaceuticals. The aim of the present study was to compare the expression of selected BBB related genes including tight junction proteins, solute carriers (SLC), ABC transporters, metabolic enzymes and to describe the paracellular properties of nine different culture models. To establish a primary BBB model rat brain capillary endothelial cells were co-cultured with rat pericytes and astrocytes (EPA). As other BBB and surrogate models four brain endothelial cells lines, rat GP8 and RBE4 cells, and human hCMEC/D3 cells with or without lithium treatment (D3 and D3L), and four epithelial cell lines, native human intestinal Caco-2 and high P-glycoprotein expressing vinblastine-selected VB-Caco-2 cells, native MDCK and MDR1 transfected MDCK canine kidney cells were used. To test transporter functionality, the permeability of 12 molecules, glucopyranose, valproate, baclofen, gabapentin, probenecid, salicylate, rosuvastatin, pravastatin, atorvastatin, tacrine, donepezil, was also measured in the EPA and epithelial models. Among the junctional protein genes, the expression level of occludin was high in all models except the GP8 and RBE4 cells, and each model expressed a unique claudin pattern. Major BBB efflux (P-glycoprotein or ABCB1) and influx transporters (GLUT-1, LAT-1) were present in all models at mRNA levels. The transcript of BCRP (ABCG2) was not expressed in MDCK, GP8 and RBE4 cells. The absence of gene expression of important BBB efflux and influx transporters BCRP, MRP6, -9, MCT6, -8, PHT2, OATPs in one or both types of epithelial models suggests that Caco-2 or MDCK models are not suitable to test drug candidates which are substrates of these transporters. Brain endothelial cell lines GP8, RBE4, D3 and D3L did not form a restrictive paracellular barrier necessary for screening small molecular weight pharmacons. Therefore, among the tested culture models, the primary cell-based EPA model is suitable for the functional analysis of the BBB.

## Introduction

The development and introduction of novel neuropharmaceuticals lags behind other groups of medicines, which is partially due to the poor central nervous system (CNS) pharmacokinetics (Banks, 2016). One of the reasons for the low number of CNS active drugs in clinical use is the restricted penetration of most drugs across the blood-brain barrier (BBB; Pardridge, 2015). The BBB is the major barrier of the CNS and is composed of brain capillary endothelial cells surrounded by pericytes embedded in the capillary basal membrane, and astrocytic endfeet (Abbott, 2013). The four main mechanisms at the level of the BBB to limit drug transport are: (i) the restricted paracellular pathway regulated by interendothelial tight junctions (TJ); (ii) the low level of non-specific transendothelial vesicular traffic; (iii) active efflux transporters which deliver metabolites from brain to blood and prevent the entry of xenobiotics and drugs to the CNS; and (iv) enzymes which metabolize drug molecules (Deli, 2011; Banks, 2016).

There is a need for reliable methods in drug development to screen drug candidates for BBB penetration, on the one hand, and to determine if substances acting in the periphery do not cross the BBB to avoid CNS side effects, on the other hand. Models that predict brain penetration are also valuable tools to study and develop new targeted nanoparticles that cross the BBB (Veszelka et al., 2015). There are several types of models for BBB permeability from *in silico* approaches to *in vivo* studies (Vastag and Keseru, 2009; Veszelka et al., 2011; Avdeef et al., 2015).

An important novel field in BBB research is the use of microfluidic devices and organ-on-chip models. These chip devices with the possibility of fluid flow provide more realistic and physiological culture conditions. In contrast to static culture inserts, in dynamic *in vitro* BBB models the endothelial cells are exposed to shear stress, induced by fluid flow, an important regulator of barrier function (Cucullo et al., 2011). In dynamic models higher transendothelial electrical resistance (TEER) and lower permeability were reported in comparison to culture insert based* in vitro* models (Cucullo et al., 2013; Booth and Kim, 2014; Walter et al., 2016). Despite these advantages, dynamic *in vitro* models have not been widely accepted for BBB permeability screening in the pharmaceutical industry yet. None of the existing dynamic *in vitro* BBB models utilizing channel microfluidics (Griep et al., 2013; Prabhakarpandian et al., 2013; Booth and Kim, 2014; Walter et al., 2016) or hollow fiber cartridges (Cucullo et al., 2011, 2013) have been assessed for a set of CNS penetrating and non-penetrating drugs with different chemical properties to elucidate a translational standard for permeability.

Cell culture BBB models are versatile tools in both basic research and permeability testing of drugs (Deli et al., 2005; Veszelka et al., 2011; Helms et al., 2016). A large number of models were developed based on primary cultures of cerebral endothelial cells or immortalized cell lines (Deli et al., 2005; Veszelka et al., 2011; Helms et al., 2016). Among the brain endothelial cell lines, the rat GP8 (Greenwood et al., 1996) and RBE4 (Roux et al., 1994), and human hCMEC/D3 cells (Weksler et al., 2005) are the best characterized and the most widely used in BBB research. RBE4 rat brain microvessel endothelial cells were employed for drug transport studies, while no drug permeability data were published for GP8 cells (Veszelka et al., 2011). The most studied BBB cell line, hCMEC/D3, is also used in drug transport and uptake experiments (Weksler et al., 2013). While the paracellular barrier is not strong in hCMEC/D3 cells, treatment with LiCl, a Wnt/β-catenin pathway activator increases TJ protein expression and barrier function (Weksler et al., 2013). The most complex *in vitro* BBB models are based on primary cultures of brain capillary endothelial cells from bovine (Dehouck et al., 1990), rat (Szabó et al., 1997), or porcine brain (Hoheisel et al., 1998; Patabendige et al., 2013), which are used in many models in co-culture with astrocytes and/or pericytes (Nakagawa et al., 2009). A recent article gives an updated overview on culture models of the BBB with guidelines for their use in permeability studies (Helms et al., 2016). A big advantage of primary BBB models is that they are complex and retain many of the *in vivo* physiological characteristics of the BBB. However, as compared to cell line models, they are more expensive, their preparation requires more time and technical expertise (Avdeef et al., 2015).

Since cost and test capacity are important factors in industrial drug screening, models based on epithelial cell lines are still used to predict permeability of CNS drug candidates (Vastag and Keseru, 2009). The most widespread culture model of human drug absorption is the Caco-2 human intestinal epithelial cell line derived from a colon adenocarcinoma, which is used primarily as a screening tool for small intestine absorption (Artursson et al., 2001). For passive diffusion compounds, Caco-2 cells give a good correlation even when compared with BBB models (Garberg et al., 2005; Hellinger et al., 2012). Caco-2 cells treated with vinblastine (VB-Caco-2), express a higher level of P-glycoprotein and this model is a good predictor of ligands for efflux transporters (Hellinger et al., 2010). The other cell lines used in pharma industry for testing drug penetration are the Madin-Darby canine kidney (MDCK) cell line and MDCK-MDR1, a subclone transfected with the human MDR1 gene. Using passive diffusion drugs MDCK cells gave a weaker correlation as compared to BBB or Caco-2 models but in the case of efflux transporter ligands the MDCK-MDR model gave accurate prediction (Garberg et al., 2005). Both Caco-2 and MDCK cells form a tight paracellular barrier and overexpress P-glycoprotein efflux pump, two factors participating in BBB permeability regulation, therefore these epithelial models in addition to prediction of intestinal absorption are also used as surrogate models for the prediction of brain penetration (Vastag and Keseru, 2009) despite cytoarchitectural differences and other dissimilarities from BBB models (Hellinger et al., 2012).

The paracellular tightness of the various BBB models measured by TEER and permeability (P_e_/P_app_) for marker molecules is in general well characterized (Deli et al., 2005; Helms et al., 2016). With the exception of P-glycoprotein, much less is known about the efflux transporter expression pattern and functionality in these models. Solute carriers (SLC) are present at the BBB in high number, where they participate in shuttling nutrients across brain endothelial cells (for review see Campos-Bedolla et al., 2014). Despite their importance, SLC expression and functionality in BBB models is understudied (Helms et al., 2016). Even less is known about phase I and II drug metabolizing enzymes in BBB culture models, with the exception of hCMEC/D3 cells (Dauchy et al., 2009).

In our previous study, we compared a primary cell-based BBB model in which rat brain endothelial cells were co-cultured with pericytes and astrocytes (EPA model, Nakagawa et al., 2009) with Caco-2, VB-Caco-2 and MDCK-MDR1 epithelial cell models provided and highlighted differences in cellular morphology, paracellular tightness and drug transport (Hellinger et al., 2012).

The aim of the present study was to extend these observations with comparative data on the expression of selected BBB related genes including TJ proteins, SLC and ABC transporters and metabolic enzymes in nine different culture models. In addition to EPA and epithelial models we also examined rat (GP8, RBE4) and human brain endothelial cell lines (D3 and D3L). To test SLC functionality the permeability of eleven drugs was also measured in the EPA and epithelial models.

## Materials and Methods

### Animals

For primary cultures of brain endothelial cells and pericytes brains were obtained from 3-week old, for glial cell culture from 2-day old Wistar outbred rats. Organ removals were performed following the regulations of the 1998. XXVIII. Hungarian law and the EU Directive 2010/63/EU about animal protection and welfare. Approval for animal studies was obtained from the local animal health authority, the Governmental Office for Csongrád County, Directorate of Food Chain Safety and Animal Health (Permit numbers: XVI./03835/001/2006, XVI./834/2012). Animals were fed on standard rodent chow and water *ad libitum* and kept under a 12 h light/dark cycle in the conventional animal house of the Biological Research Centre, Szeged.

### Cell Cultures

Isolation of primary rat brain endothelial cells, glia and pericytes and the construction of the *in vitro* BBB model were performed according to the method described in our previous studies (Nakagawa et al., 2009; Walter et al., 2015). After isolation, cells were seeded on Petri dishes coated with 100 μg/ml collagen type IV and 100 μg/ml fibronectin in sterile distilled water. Brain endothelial cells were cultured in DMEM/ HAM’s F-12 (Gibco, Life Technologies, Carlsbad, CA, USA), 15% plasma-derived bovine serum (PDS, First Link, Wolverhampton, UK), 100 μg/ml heparin, 5 μg/ml insulin, 5 μg/ml transferrin, 5 ng/ml sodium selenite, 1 ng/ml basic fibroblast growth factor (bFGF, Roche, Basel, Switzerland) and 50 μg/ml gentamicin. During the first 3 days of culture the medium of brain endothelial cells contained 3 μg/ml puromycin to eliminate P-glycoprotein negative, contaminating cell types (Perrière et al., 2005). Primary rat brain pericytes were isolated using the same method as for brain endothelial cells, except that pericytes were plated onto uncoated Petri dishes (Orange Scientific, Braine-l’Alleud, Belgium). Primary cultures of rat glial cells were prepared from one-day-old Wistar rats (Perrière et al., 2005) and passaged to 10 cm Petri dishes (Corning, Costar, New York, NY, USA) coated with 100 μg/ml collagen type IV in sterile distilled water and cultured for 2 weeks before use for the triple co-culture model. Pericytes and glial cells were cultured in DMEM/HAM’s F-12 supplemented with 10% fetal bovine serum (FBS, Pan-Biotech GmbH) and 50 μg/ml gentamicin. For the triple co-culture model, pericytes (P3) were passaged to the bottom side of tissue culture inserts with 75 mm diameter (Transwell 3419, polycarbonate membrane, 0.4 μm pore size, Corning Costar) at a density of 1.5 × 10^4^ cells/cm^2^. Endothelial cells were seeded to the upper side of the membranes (7.5 × 10^4^ cells/cm^2^) and placed to 10 cm Petri dish containing glial cells. Both compartments received endothelial culture medium and the three types of cells were cultured together for 4 or 5 days (Nakagawa et al., 2009; Walter et al., 2015). In transport assays the triple culture BBB model was prepared on 12 well plate Transwell inserts (polyester membrane, 0.4 μm pore size, Corning Costar). When brain endothelial cell layers became confluent 550 nM hydrocortisone was added to tighten the junctions (Deli et al., 2005).

GP8 rat brain endothelial cell line (provided by Dr. John Greenwood, University College London, UK) was cultured in DMEM/ HAM’s F-12, 15% PDS, 100 μg/ml heparin, 1 ng/ml bFGF and 50 μg/ml gentamicin.

RBE4 rat brain endothelial cell line (provided by Dr. Pierre-Olivier Couraud, Institut Cochin, Paris, France) was grown in DMEM/ HAM’s F-12, supplemented with 10% FBS, 1 ng/ml bFGF and 50 μg/ml gentamicin.

Cultures of human brain endothelial hCMEC/D3 cell line (≤passage number 35) were grown in MCDB 131 medium (Pan Biotech) supplemented with 5% FBS, GlutaMAX (100×, LifeTechnologies, Carlsbad, CA, USA), lipid supplement (100×, Life Technologies, Carlsbad, CA, USA), 10 μg/ml ascorbic acid, 550 nM hydrocortisone, 100 μg/ml heparin, 1 ng/ml basic fibroblast growth factor (bFGF, Roche, USA), 2.5 μg/ml insulin, 2.5 μg/ml transferrin, 2.5 ng/ml sodium selenite and 50 μg/ml gentamicin (Weksler et al., 2005). For differentiation of this cell line (D3L group), the medium was supplemented with 10 mM lithium chloride (LiCl) at the first change of medium (Paolinelli et al., 2013). For the three brain endothelial cell lines, we used culture media with very similar or identical composition to those that were originally described for their growth and maintenance.

Human Caco-2 intestinal epithelial cell line (ATCC cat.no. HTB-37) was maintained in DMEM/HAM’s F-12 culture medium supplemented with 10% FBS and 50 μg/ml gentamicin. VB-Caco-2 cultures were created from Caco-2 cultures by selecting cells with 10 nM vinblastine treatment for at least six passages (Hellinger et al., 2010). Treatment leads to a more homogeneous cell morphology and a higher expression level of efflux pumps.

Parent and MDR1 transfected Madin-Darby canine kidney epithelial cells (Evers et al., 2000) were obtained from the Netherlands Cancer Institute (Amsterdam, Netherlands). The tissue culture medium consisted of 4.5 g/l glucose containing DMEM supplemented with 10% FBS, penicillin (50 units) and streptomycin (0.05 mg/ml). For RNS isolation, all cell lines were seeded in 10 cm Petri dishes and for transport assays cells were passaged onto 12-well plate Transwell inserts coated with 0.05% rat tail collagen in sterile distilled water.

### Transendothelial Electrical Resistance Measurement

Transendothelial electrical resistance (TEER), reflecting the permeability of TJ for sodium ions, was measured by an EVOM voltohmmeter (World Precision Instruments, Sarasota, FL, USA) combined with STX-2 electrodes. Recorded resistance was expressed to the surface area of the filters (Ω × cm^2^, Transwell inserts, polystyrene membrane, 0.4 μm pore size, Corning Costar, USA). TEER of cell-free inserts (110 Ω × cm^2^) was subtracted from the measured data.

### Permeability Measurement

For permeability tests the epithelial and endothelial cell types were cultured on culture inserts (polycarbonate membrane, 0.4 μm pore size, 1.2 cm^2^ surface, Transwell, Corning Costar). The inserts were transferred to 12-well plates containing 1.5 ml Ringer-Hepes buffer (EPA model) or HBSS-Hepes buffer (epithelial models) in the lower (basal/abluminal) compartments. In the upper (apical/luminal) compartments culture medium was replaced by 0.5 ml buffer containing permeability marker molecules albumin (1 mg/ml; Mw: 65 kDa) labeled with Evans blue (167.5 μg/ml) and fluorescein (10 μg/ml; Mw: 376 Da). After incubation with permeability markers for 30 min, samples were collected from both compartments and concentrations of the marker molecules were determined by a fluorescence multi-well plate reader (Fluostar Optima; excitation wavelength: 485 nm, emission wavelength: 535 nm in the case of fluorescein and excitation wavelength: 584 nm, emission wavelength: 680 nm in the case of Evans-blue labeled albumin). The apparent permeability coefficients (P_app_) were calculated as we described previously (Kürti et al., 2012). Briefly, cleared volume was calculated from the concentration difference of the tracer in the lower/basal compartment (Δ[C]_B_) after 30 min (t) and upper/apical compartments at 0 h ([C]_A_), the volume of the lower/basal compartment (V_B_; 1.5 mL) and the surface area available for permeability (A; 1.1 cm^2^) by the following equation:

$${\text{P}}_{\text{app}}(\text{cm/s})\text{= }\frac{{\left[C\right]}_{\text{B}}\times V_{\text{B}}}{A\times {\left[C\right]}_{\text{A}}\times t}$$

For the permeability study on the EPA and epithelial models, nine different SLC ligands (at 10 μM concentrations, except glucopyranose (100 μM) and valproic acid (300 μM), 60–120 min) and two anticholinergic drugs, tacrine and donepezil (0.5 μM, 30 min), approved for the treatment of Alzheimer’s disease, were tested. The transport of tacrine and donepezil was also measured in the presence of choline and carnitine, two endogenous cationic metabolites to examine whether they share influx transporters. The concentrations of the test molecules in samples from the donor and acceptor compartments were determined by high-pressure liquid chromatography (HPLC) or liquid chromatography-mass spectrometry (LC/MS). P_app_ was calculated for each drug as described above. The efflux or permeability directional ratio (PDR) was given as the ratio of P_app_ values in BA to AB direction (Hellinger et al., 2012).

### Immunohistochemistry

Brain endothelial and epithelial cells were cultured on rat tail collagen coated glass cover slips. Endothelial cells were stained for the integral membrane tight junction protein claudin-5 and epithelial cells were stained for claudin-4. After the removal of the culture medium cells were fixed with ethanol—acetic acid (95:5 mixture) for 5 min at −20°C, blocked with 3% bovine serum albumin diluted in phosphate buffer (PBS) and incubated overnight with primary antibodies: anti-claudin-5 (rabbit polyclonal antibody, 1:200, Sigma, AB_10753223) or anti-claudin-4 (mouse monoclonal antibody, 1:200, Thermo Fisher Scientific, AB_2533096). Incubation with secondary antibodies Cy3-labeled anti-rabbit (Sigma) or Alexa488-labeled anti-mouse immunglobulin (Invitrogen, Life Technologies, 1:500) and Hoechst dye 33342 (Sigma) for nucleus staining lasted for 1 h. Cells were washed three times with PBS between incubations. After mounting the samples (Fluoromount-G; Southern Biotech, Birmingham, AL, USA) staining was visualized by Olympus Fluoview FV1000 confocal laser scanning microscope (Olympus Life Science Europe GmbH, Hamburg, Germany).

### RNA Isolation and Quality Control

The endothelial (primary brain endothelial cells, GP8, RBE4, D3, D3L) and epithelial cells (Caco-2, VB-Caco-2, MDCK and MDCK-MDR1) were cultured for 5 days in 10 cm dishes. After reaching confluency cells were scraped, collected and cell pellets were used for total RNA isolation using RNAqueous-4PCR Kit (Ambion, Life Technologies, Austin, TX, USA) with DNase1 (RNase-free) treatment according to the manufacturer’s instructions. The concentrations and purity of the DNase-treated RNA samples were assessed by a NanoDrop ND-1000 spectrophotometer (NanoDrop Technologies, Rockland, DE, USA). The integrities of the isolated RNAs were characterized using Bioanalyzer 2100 (Agilent Technologies, Santa Clara, CA, USA). The RNA integrity numbers (RIN) were between 9.2 and 10 in the case of all studied RNA samples.

### Quantitative Real-Time Polymerase Chain Reaction and Data Analysis

In all cases, cDNA synthesis was performed on 1 μg total RNA samples by a High Capacity cDNA Reverse Transcription Kit (Life Technologies) using random hexanucleotide primers and MultiScribe Reverse Transcriptase in the presence of RNase inhibitor according to the manufacturer’s protocols. The expression of the selected BBB genes was analyzed by quantitative PCR using TaqMan Low Density Array 384-well microfluidic cards preloaded with TaqMan Gene Expression Assays (Life Technologies). The list of the studied genes and applied TaqMan Gene Expression Assays are shown in the supplementary materials (Supplementary Table S1). Quantitative real-time PCRs (qPCR) were performed by ABI TaqMan Universal Master Mix (Life Technologies) using the ABI Prism 7900 system (Applied Biosystems, Life Technologies). qPCR data were analyzed using the ABI SDS 2.0 software (Applied Biosystems, Life Technologies). In all samples the expression of genes was normalized to 18S rRNA, which was used as an endogenous control (ΔC_t_ = C_tgene_ − C_t18S rRNA_). Expression values of studied genes were determined based on the normalized expression of genes calculated with 2^−ΔC_t_^ formula which were correlated to the lowest normalized expression measured by the applied qPCR method. For quantification of the relative expression level of genes of interest, the normalized expression data were analyzed using the comparative ΔΔC_t_ method (Livak and Schmittgen, 2001; Tóth et al., 2014).

### Statistical Analysis

Data are presented as means ± SEM or SD. Values were compared using unpaired *t*-test, one-way or two-way analyses of variances following Dunnett or Bonferroni multiple comparison posttests (GraphPadPrism 5.0; GraphPad Software, USA). Changes were considered statistically significant at *P* < 0.05. All experiments were repeated at least two times and the number of parallel samples was 4–8.

## Results

### Comparison of the Triple Co-culture BBB Model With Caco-2, VB-Caco-2, MDCK and MDCK-MDR1 Epithelial Cell Lines: Expression of Selected Tight Junction Protein, Transporter and Metabolic Enzyme Genes

#### Tight Junction Proteins

**Figure 1:**
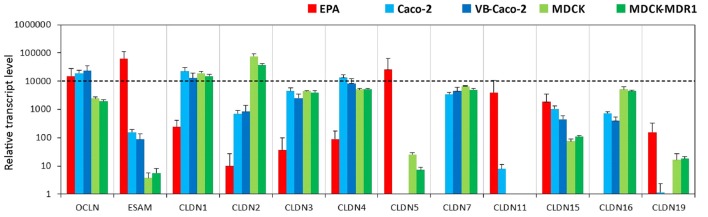
Relative transcript levels of selected genes encoding tight junction proteins measured by inventoried TaqMan Gene Expression Assays in a primary rat brain endothelial cell-based blood-brain barrier (BBB) model (EPA) and in epithelial cell line models (Caco2, VB-Caco2, MDCK and MDCK-MDR1).

Primary rat brain endothelial cells grown in co-culture with glial cells and pericytes (EPA) produced high levels of mRNA for key tight junction proteins such as claudin-5 (CLDN5), occludin and the endothelial cells specific adhesion molecule ESAM (Figure 1). Caco-2 epithelial cells also showed a high level of expression for occludin, while in MDCK cells it was lower as compared to both EPA and Caco-2 models (Supplementary Figure S1). The gene of endothelial cell specific ESAM was expressed at a low level in epithelial cells. High level of CLDN5 expression, comparable to occludin and ESAM, was measured in brain endothelial cells in the EPA model. In the BBB model, the cells expressed low level of CLDN1, CLDN2, CLDN3, CLDN4 transcripts and did not express CLDN7 mRNA, specific for epithelial cells. The absence of CLDN16 transcript and relatively high level of CLDN15 mRNA could be observed in the rat primary BBB model (Figure 1). CLDN19 mRNA was detected in the rat BBB model, but not in Caco-2 epithelial cells. The expression pattern of claudins in MDCK cells was similar to that of the Caco-2 cells with some exceptions, CLDN2, CLDN5, CLDN16 and CLDN19 gene expression levels were higher in the kidney epithelial cells as compared to the intestinal epithelial cell lines (Figure 1).

#### Solute Carrier or Other Transporters

**Figure 2:**
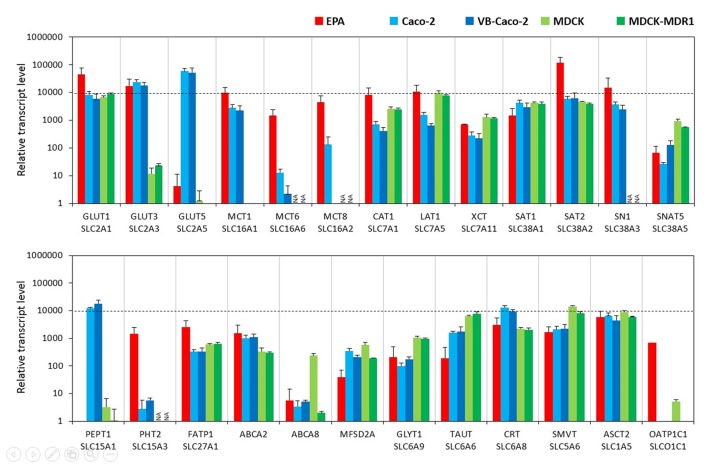
Relative transcript levels of selected genes encoding solute carriers (SLC) and other nutrient transporters measured by inventoried TaqMan Gene Expression Assays in a primary rat brain endothelial cell-based BBB model (EPA) and in epithelial cell line models (Caco-2, VB-Caco-2, MDCK and MDCK-MDR1). NA: assay not available.

In the BBB model, primary brain endothelial cells expressed high levels of mRNAs for glucose transporter GLUT1 (Slc2a1) and GLUT3 (Slc2a3; Figure 2) and the transcript level of GLUT5 (Slc2a5) was low. Caco-2 epithelial cells expressed high levels of all three GLUT transporters from which GLUT5 showed the highest and GLUT1 the lowest expression (Supplementary Figure S2). MDCK cells also expressed high levels of the GLUT1 gene, a low level of GLUT3, and did not express GLUT5. Brain endothelial cells in co-culture expressed high amount of mRNA coding monocarboxylic acid transporters MCT1 (Slc16a1), -2 (Slc16a2) and -6 (Slc16a6), which provide secondary energy sources and thyroid hormones for the CNS, respectively (Figure 2). Caco-2 epithelial cells expressed a high level of MCT1 only and a lower level of MCT8 and MCT6 mRNAs. MDCK cells did not produce the MCT1 transcript. In the BBB model, high mRNA expression levels were measured for all the amino acid transporters tested except for small neutral amino acid transporter SNAT5 (Slc38a5), where a moderate expression level was measured. Caco-2 and MDCK epithelial cells also expressed all these transporter genes at a high and moderate level. Among the peptide transport systems tested, cells of the primary rat brain endothelial cell-based model did not express PEPT1 (Slc15a1) but produced a significant amount of PHT2 transcript (Slc15a3; Figure 2). In contrast, in the Caco-2 cells the expression level of PEPT1 was relatively high, but low in the case of PHT2. MDCK cell lines produced a small amount of PEPT1 mRNA. Fatty acid transporter FATP1 (Slc27a1) was well expressed in the BBB and the epithelial models (Figure 2). Among the sterol transporters, the ABCA2 gene was expressed in all models, while the highest transcript level of ABCA8 was found in MDCK cells, however, in the other investigated models its transcript level was low. The gene of MFSD2A, a transporter for ω-3 fatty acids was also transcribed in all five models. Its expression level was higher in the Caco-2 and MDCK cells as compared to the EPA model (Figure 2). Among the genes of Slc6 family rat brain endothelial cells produced a high amount of mRNA of the gene of the carrier for creatine (CRT, Slc6a8), and moderate transcript levels were measured for glycine (GLYT1, Slc6a9) and taurine (TAUT, Slc6a6) genes (Figure 2). A similar expression pattern was seen for Caco-2 and MDCK epithelial cells. High levels of mRNAs of the sodium-dependent vitamin transporter (SMVT, Slc5a6) and vitamin C transporters ASCT2 (Slc1a5) and ASCT1 (Slc1a4; Supplementary Table S2) genes were detected in rat brain endothelial cells (Figure 2). Epithelial cells also expressed all these carriers. Except for the absence of dopamine transporter gene transcription (DAT, Slc6a3), low transcript levels of the noradrenalin (NET, Slc6a2), serotonin (SERT, Slc6a4), and GABA (GAT1, Slc6a1; GAT2, Slc6a13; GAT3, Slc6a11) genes were seen in the BBB model (Supplementary Table S2). Epithelial cells did not produce a detectable amount of mRNAs of the genes of neurotransmitter carriers except for the high-level expression of the SERT gene in Caco-2 cells (Supplementary Table S2). The genes of organic anion-transporting polypeptides (OATP1C1, Slco1c1; OATP1A2, Slco1a2) which mediate the transport of thyroid and steroid hormones in addition to organic anions, were expressed in primary brain endothelial cells but not in epithelial models (Figure 2 and Supplementary Table S2).

#### Efflux Transporters

**Figure 3:**
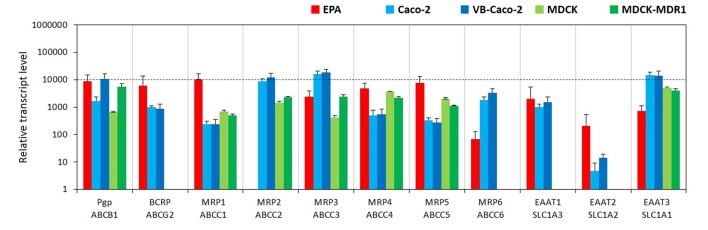
Relative transcript levels of selected genes encoding efflux transporters measured by inventoried TaqMan Gene Expression Assays in a primary rat brain endothelial cell-based BBB model (EPA) and in epithelial cell line models (Caco-2, VB-Caco-2, MDCK and MDCK-MDR1).

The BBB model EPA expressed a similarly high amount of mRNA for the two primary efflux transporters at the BBB, P-glycoprotein (Pgp, ABCB1) and breast cancer resistance protein (BCRP, ABCG2; Figure 3). Vinblastine—selected VB-Caco-2 cells showed significantly higher P-gp expression than native Caco-2 cells, while MDCK-MDR1 cells transfected with human ABCB1 gene also produced a higher level of canine ABCB1 gene transcript (Figure 3, Supplementary Figure S3). The expression of the BCRP gene could not be detected in the kidney epithelial cells, but Caco-2 cells do express BCRP. Among the tested multidrug resistance-associated proteins (MRP), the mRNAs of the MRP-1, -3, -4 and -5 were the four most dominant efflux transporter transcripts in brain endothelial cells, the MRP-6 genes were expressed at a lower level, while the MRP-2 gene was not expressed at all (Figure 3). Caco-2 cells produced high amounts of mRNAs of the MRP-2, -3 and -6, and a lower level of MRP-1, -4 and -5 genes. MDCK cells had a similar expression pattern to that of Caco-2 cells, except that they did not transcribe the MRP-6 gene. Among the excitatory amino acid transporters (EAAT, Slc1a family) which are participating in the efflux transport of glutamate across the BBB and are responsible for the low level of glutamate in the brain interstitial fluid, the EAAT1 (Slc1a3) gene was highly expressed in brain endothelial cells (Figure 3). The EAAT2 (Slc1a2) and EAAT3 (Slc1a1) genes were expressed at moderate levels. In Caco-2 and MDCK cells the expression level of EAAT3 gene was the highest. The gene of EAAT1 was also highly expressed in Caco-2 cells, but not expressed in MDCK cells. In contrast to the EPA model the transcript level of the EAAT2 gene was very low in epithelial cell lines.

#### Metabolic Enzymes

**Figure 4:**
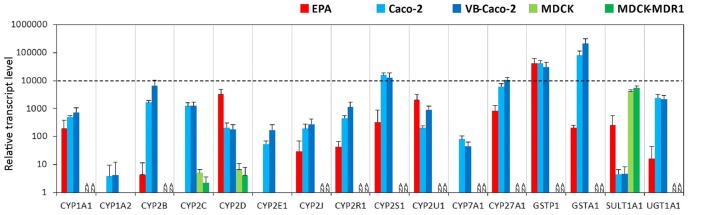
Relative transcript levels of selected genes encoding metabolic enzymes measured by inventoried TaqMan Gene Expression Assays in a primary rat brain endothelial cell-based BBB model (EPA) and in epithelial cell line models (Caco-2, VB-Caco-2, MDCK and MDCK-MDR1). NA: assay not available.

Among the tested genes of the phase-I drug metabolic enzymes, brain endothelial cells of the EPA BBB model expressed mRNA of CYP1A1, CYP2D6, CYP2R1, CYP2S1, CYP2U1 and CYP27A1 cytochrome P450 enzymes (Figure 4, Supplementary Figure S4), but not CYP1A2, CYP2E1 and CYP7A1. In Caco-2 cells the highest transcript level of the cytochrome P450 genes was seen for CYP2S1 and CYP27A1 genes. Only two genes, the CYP2C9 and CYP2D6 genes were transcriptionally active in MDCK cells. The other genes either could not be tested in MDCK cells due to the unavailability of appropriate gene probes or they were not expressed. Among the genes of phase-II metabolic enzymes selected, rat brain endothelial cells expressed high levels of mRNA of the glutathione S-transferase π (GSTP1 gene; Figure 4). Caco-2 cells expressed a high level of both GSTP1 and GSTA1 genes of the glutathione S-transferase enzyme. The expression level of the sulfotransferase 1A1 (SULT1A1) gene was low in Caco-2 cells, moderate in EPA and high in the MDCK cell lines. The expression level of the drug metabolizing enzyme UDP-glucuronosyltransferase UGT1A1 gene was moderate in the EPA model and high in Caco-2 cells (Figure 4). The relative expression levels of all the measured genes in the BBB and the epithelial models are shown in Supplementary Table S2.

### Comparison of the Triple Co-culture BBB Model With GP8, RBE4 and D3 Brain Endothelial Cell Lines: Expression of Selected Tight Junction Protein, Transporter and Metabolic Enzyme Genes

#### Tight Junction Proteins

**Figure 5:**
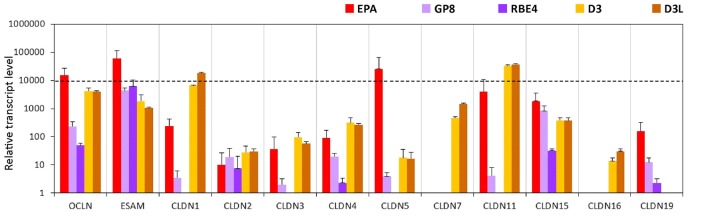
Relative transcript levels of selected genes encoding tight junction proteins measured by inventoried TaqMan Gene Expression Assays in a primary rat brain endothelial cell-based BBB model (EPA) and in brain endothelial cell line models (GP8, RBE4, D3 and D3L).

The expression level of occludin is high in the EPA and the D3 models but low in the rat GP8 and RBE4 cell lines (Figure 5, Supplementary Figure S5). The endothelial cell specific adhesion molecule (ESAM) was expressed at high levels in all brain endothelial cell models and was the highest in primary rat brain endothelial cells. The level of CLDN5 mRNA was significantly higher in the primary EPA model than in any of the cell lines (Figure 5). The expression of the CLDN5 gene in the RBE4 cell line was below the detection limit. Among the claudins, the expression of the epithelial specific CLDN2 and -4 genes, was lower in the endothelial than in the epithelial models, while CLDN7 and CLDN16 was not expressed in the rat BBB models, only in the human D3 cells (Figure 5). The CLDN1 and CLDN11 genes were expressed in the EPA and D3 models, their transcript levels were low in GP8 cells and absent in RBE4 cells. With the exception of ESAM, occludin and CLDN15 all of the tested TJ protein genes were not or expressed at low level in the rat GP8 and RBE4 cell lines. The lithium treatment did not change the transcript profile of the TJ protein genes in the D3L model compared to the untreated D3 cells.

#### Solute Carriers and Other Transporters

**Figure 6:**
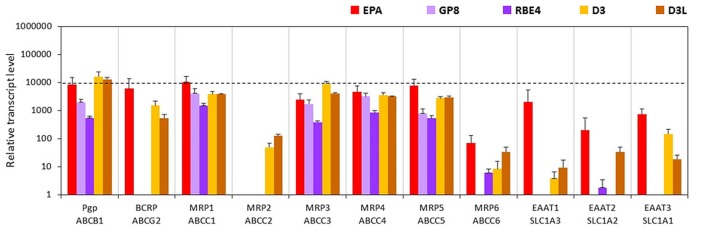
Relative transcript levels of selected genes encoding SLC and other nutrient transporters measured by inventoried TaqMan Gene Expression Assays in a primary rat brain endothelial cell-based BBB model (EPA) and in brain endothelial cell line models (GP8, RBE4, D3 and D3L).

The expression levels of glucose transporters GLUT1 and -3 genes were the highest in the EPA model. These two carriers were also well transcribed in the other four cell lines, except for GLUT3 in GP8 cells. In the D3 and GP8 models the primary glucose transporter was GLUT1, while in RBE4 cells it was GLUT3 (Figure 6, Supplementary Figure S6). The mRNA level of the GLUT5 gene was low in all tested endothelial models, and not expressed in RBE4 cells. The monocarboxylic acid carrier MCT1 gene was also well expressed in all brain endothelial cells except for RBE4. The MCT8 expression was the highest in the EPA model. The MCT6 gene expression was at a moderate level in all cell types.

From the seven amino acid transporters examined, high expression level was measured for CAT1, LAT1 and SAT2 genes in all models. In the human D3 cells, the expression of XCT, SAT1 and SNAT5 genes was higher than in the rat BBB models. The SN1 transcript level was high in the EPA model and moderate in D3 cells. GP8 and RBE4 rat brain endothelial cell lines expressed low level of SN1 and did not express SNAT5. From the two tested peptide transporters PEPT1 was not expressed in brain endothelial models (Figure 6). The PHT2 gene expression level was moderate in the EPA and D3 models and low in GP8 and RBE4 cells.

The fatty acid transporter FATP1 gene was equally well expressed in all BBB models. From the lipid transporters, the ABCA2 gene was expressed in all models at a moderate level. The highest mRNA level of ABCA8 was measured in D3 and D3L cells. In the EPA model, the ABCA8 gene expression was low and in GP8 and RBE4 cell lines it was not expressed. The highest expression of the MFSD2A gene coding for the CNS transporter for docosahexaenoic acid was measured in the D3 cells. The transcription MFSD2A occurred at a moderate level in the EPA model, but not in GP8 and RBE4 cells (Figure 6).

From the SLC6 family, moderate expression levels were measured for the genes of creatine (CRT), glycine (GLYT1) and taurine (TAUT) carriers in all brain endothelial cells (Figure 6) except of the TAUT gene, which was expressed at a low level in GP8 and RBE4 cells. The genes of the vitamin transporters, SMVT and ASCT2 were well expressed in all endothelial models, only the ASCT2 mRNA level was low in GP8 cells (Figure 6). The gene of the organic anion-transporting polypeptide OATP1C1 was expressed only in the primary EPA model but not in the brain endothelial cell lines. Lithium treatment in D3 cells elevated the expression level in half of the tested carriers and transporter genes (GLUT3, -5, MCT8, SN1, SNAT5, PEPT1, FATP1, ABCA2, GLYT1, TAUT, CRT, ASCT2; Figure 6).

#### Efflux Transporters

**Figure 7:**
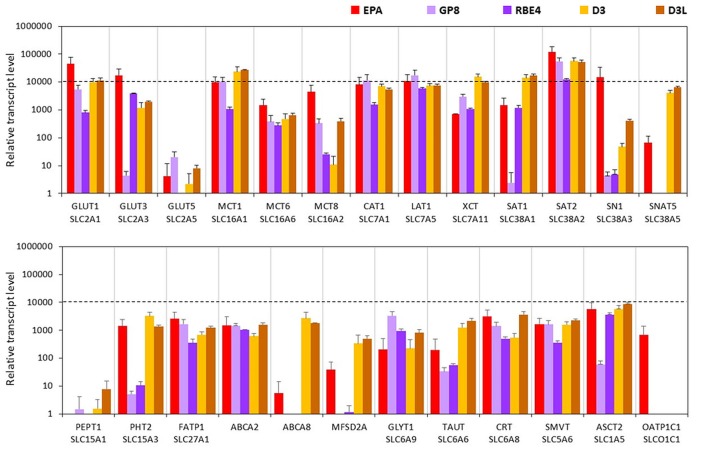
Relative transcript levels of selected genes encoding efflux transporters measured by inventoried TaqMan Gene Expression Assays in a primary rat brain endothelial cell-based BBB model (EPA) and in brain endothelial cell line models (GP8, RBE4, D3 and D3L).

The gene of one of the main efflux transporters of the BBB, Pgp was well expressed in both the EPA (Abcb1a, Figure 7; Abcb1b, Supplementary Table S3) and the D3 models. The BCRP gene expression was also high in the EPA but lower in the D3 models. Compared to the EPA and D3 models, the expression level of the Abcb1a gene was lower in the GP8 and RBE4 cells, while the BCRP transcript level was below the detection limit in these models (Figure 7, Supplementary Figure S7). The genes of the ABC transporter subfamily C members MRP1, 3, 4, 5 were well expressed in all models. The lowest expression for these genes was seen in RBE4 cells. MRP2 was only expressed in the D3 models. The MRP6 gene expression was the highest in the primary EPA model, very low in the other endothelial models and not detected in GP8 cells. The genes of the glutamate efflux transporters EAAT1, -2, -3 were expressed at high and moderate levels in the EPA model, lower levels in D3 cells and at a negligible level in the rat brain endothelial cell lines (Figure 7). In D3 cells a trend for elevation in gene expression was observed for MRP2, -6 and EAAT1, while an increase was seen in EAAT2 expression.

#### Metabolic Enzymes

**Figure 8:**
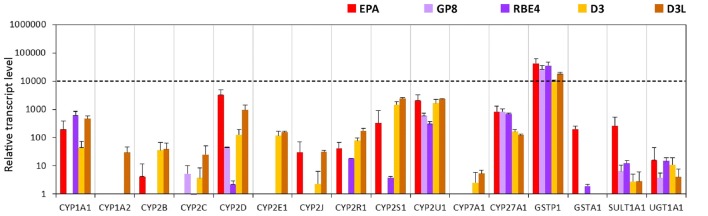
Relative transcript levels of selected genes encoding metabolic enzymes measured by inventoried TaqMan Gene Expression Assays in a primary rat brain endothelial cell-based BBB model (EPA) and in brain endothelial cell line models (GP8, RBE4, D3 and D3L).

We found two cytochrome P450 enzymes, CYP2U1 and CYP27A1 from the tested 12 isoforms which were expressed in all brain endothelial models (Figure 8, Supplementary Figure S8). No gene expression was seen for Cyp1a2, Cyp2c11, Cyp2e1 and Cyp7a1 genes in the EPA model, however these genes (CYP1A2, CYP2C9, CYP2E1, CYP7A1) were expressed in D3 cells. In the primary EPA model, the Cyp2d4, in D3 brain endothelial cells the CYP2S1 and CYP2U1 transcript levels were the highest among the genes of the selected phase I enzymes (Figure 8). GP8 cells did not express Cyp genes except for the Cyp2d4, Cyp2u1 and Cyp27a1 genes. In RBE4 cells only 3 Cyp enzyme genes, Cyp1a1, Cyp2u1 and Cyp27a1, were well expressed, while the others were expressed at very low or negligible levels.

The gene expression level of the GSTP1 phase II enzyme was high in all models (Figure 8). The GSTA1, SULT1A1 and UGT1A1 genes were expressed in the EPA and were absent or very low in the other brain endothelial models (Figure 8).

### Evaluation of the Barrier Integrity in Brain Endothelial and Epithelial Models

Among the brain endothelial cell-based models the TEER was the highest in the primary cell-based BBB model (475 ± 48 Ω cm^2^; Table 1). Both native Caco-2 (854 ± 24 Ω cm^2^) and vinblastine selected VB-Caco-2 (1186 ± 71 Ω cm^2^) models showed a tight paracellular barrier. The MDCK, MDCK-MDR1, RBE4, D3 and D3L cultures presented a TEER that was below 100 Ω cm^2^. Treatment with lithium significantly elevated the TEER of D3 cells (unpaired *t*-test, *P* < 0.0004). The lowest resistance was measured in the GP8 cell line model. The EPA and the epithelial models were the least permeable for both fluorescein, a low molecular weight marker of paracellular integrity, and for Evans blue-labeled albumin, the marker of transcellular permeability (Table 1). All the four brain endothelial cell lines demonstrated significant, one order of magnitude higher values of P_app_ for both markers (Table 1).

**Table 1:** Paracellular tightness of different brain endothelial and epithelial cell culture models measured by transendothelial/epithelial electrical resistance (TEER) and permeability for markers fluorescein and albumin.

Models	TEER (Ω × cm^2^)	Fluorescein P_app_ (10^−6^ cm/s)	Albumin P_app_ (10^−6^ cm/s)
EPA	475 ± 48	2.1 ± 0.25	0.2 ± 0.03
CaCo2	854 ± 24	1.5 ± 0.28	0.8 ± 0.09
VB-CaCo2	1186 ± 71	0.3 ± 0.06	0.2 ± 0.08
MDCK	72 ± 9	2.8 ± 0.23	0.6 ± 0.05
MDCK-MDR1	81 ± 7	2.7 ± 0.31	-
GP8	28 ± 13	39.8 ± 3.51	16.5 ± 7.51
RBE4	64 ± 5	27.4 ± 1.63	3.9 ± 0.23
D3	45 ± 2	22.2 ± 3.71	2.1 ± 0.31
D3L	86 ± 6	19.3 ± 1.22	1.7 ±0.12

*P_app_: apparent permeability coefficients. Values are presented as mean ± SD*.

**Figure 9:**
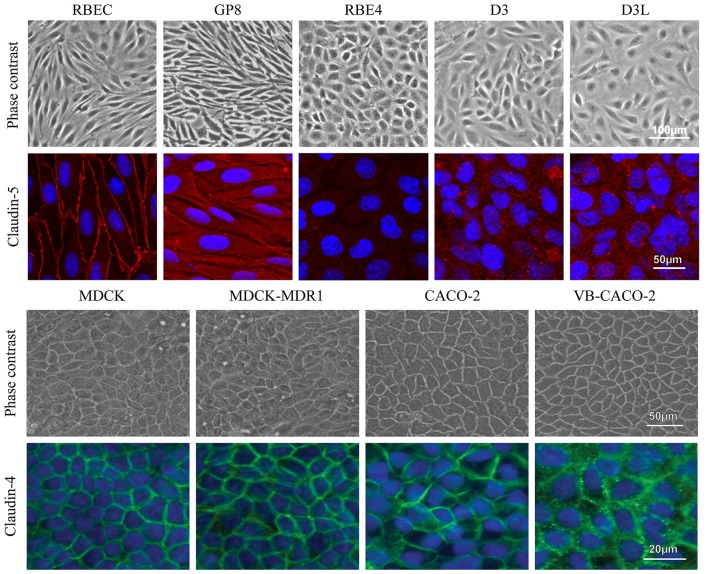
Different types of brain endothelial and epithelial cell cultures examined by phase contrast microscopy and stained for tight junction proteins CLDN5 (red) and CLDN4 (green), and cell nucleus (blue). The CLDN5 immunostaining was well visible on the cell border of RBEC cells, while it was very weak or undetectable in the brain endothelial cell lines. MDCK and Caco-2 epithelial cells showed an intense staining for CLDN4. Bars: 50 and 100 μm (phase contrast pictures); 20 and 50 μm (immunostaining pictures).

The confluent, non-overlapping, uniform monolayer of cells in all investigated models was well visible on both the phase contrast images and the immunostainings for junctional proteins (Figure 9). The most striking difference in the pattern of endothelial cells compared to epithelial cells was the cell shape. Endothelial cells were elongated, fusiform, and formed a swirling pattern well observable on Figure 9. Primary brain endothelial cells in the EPA model gave a strong pericellular staining for claudin-5, the most abundant claudin type at the BBB. Claudin-5 staining was concentrated to the cell border at the interendothelial junctions. In the brain endothelial cell lines, the claudin-5 staining was cytoplasmic and not visible at the cell border (Figure 9, Supplementary Figure S9). Epithelial cells presented characteristic “cobblestone” morphology. In accordance with the gene expression data (Figure 1) epithelial cells stained intensely for claudin-4 (Figure 9), a TJ protein typical for epithelial cells.

### SLC Related Drug Transport in the Culture Models

**Figure 10:**
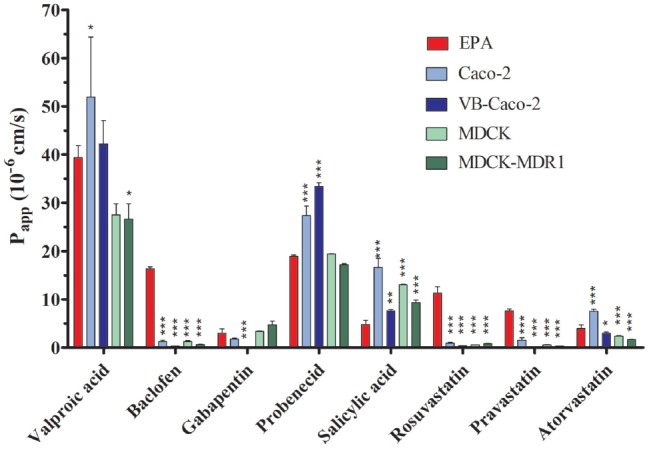
Permeability coefficients (P_app_) for selected drugs on primary rat brain endothelial cell-based BBB model (EPA) and in epithelial cell line models (Caco-2, VB-Caco-2, MDCK and MDCK-MDR1) measured in the apical to basal (blood-to-brain) direction. Statistics: mean ± SD, *n* = 4, ANOVA and Dunnett test; *P < 0.05, **P < 0.01; ***P < 0.001 compared to control.

The selected nine ligands for SLCs were tested on the BBB and the four epithelial models. Drug penetration in the apical to basal (A-B) direction (P_app_) and the PDR ratio (PDR: P_app_ B-A/P_app_ A-B) are shown in Table 2. The GLUT1 ligand 3-O-methyl-D-glucopyranose was transported in all models. PDR ratios indicating active influx was observed in the case of EPA and Caco-2 models. Among the clinically used drugs in the case of LAT1 substrates, the highest permeability values were measured for valproic acid on all models (Figure 10). Baclofen penetration was the highest in the EPA model, while it was very low across the epithelial cell layers. In this group of drugs, the lowest P_app_ was measured for gabapentin on all models. None of the LAT1 ligand drugs were identified as efflux pump ligands based on the PDR (Table 2). To further prove the functionality of LAT1, L-DOPA (3, 4-dihydroxy-L-phenylalanine) transport was measured in the EPA model in two directions. We observed a high P_app_ in A-B direction (73 ± 4 10^–6^ cm/s) and a low PDR (0.4). The second highest P_app_ value was measured for the organic anion probenecid, especially in the Caco-2 models (Table 2). The other organic anion, salicylic acid had a low permeability in the BBB model as compared with the epithelial models (Figure 10). Among the tested three statins, rosuvastatin and pravastatin had significantly higher P_app_ in the BBB model than in the Caco-2 or MDCK cells. In the statin group the lowest P_app_ value in A–B direction and the highest PDR (1.4) was measured for atorvastatin in the EPA model, indicating that atorvastatin may be the subject of active efflux transport. Parallel to the low penetration, the PDR for the statins was higher in the epithelial than in the BBB model (Table 2).

**Table 2:** Permeability coefficients (P_app_) for selected drugs on the primary rat brain endothelial cell-based BBB model (EPA) and in epithelial cell line models (Caco-2, VB-Caco-2, MDCK and MDCK-MDR1) measured in the apical to basal (A-B) direction.

Ligands	SLC	EPA		Caco-2		VB-Caco-2		MDCK		MDCK-MDR1	
		P_app_ A-B	PDR	P_app_ A-B	PDR	P_app_ A-B	PDR	P_app_ A-B	PDR	P_app_ A-B	PDR
Glucopyranose	GLUT1	<65.7	>0.14	57.83	0.64	17.17	1.17	<18.5	NA	<18.5	NA
Valproic acid	LAT1, OATPs	39.37	0.98	51.91	0.82	42.22	0.87	26.62	1.22	27.49	1.26
Baclofen	LAT1	16.34	0.85	1.23	0.77	0.31	0.47	1.28	0.77	0.68	0.82
Gabapentin	LAT1, Pgp, BCRP	2.98	0.92	1.80	0.77	<0.88	>0.27	3.35	0.28	4.75	0.42
Probenecid	MCT, OATs	18.96	0.79	27.34	0.88	33.39	0.88	19.37	0.83	17.23	0.98
Salicylic acid	MCT1, OATPs, MRP4	4.78	1.88	16.63	1.11	7.60	1.19	13.01	0.86	9.31	0.85
Rosuvastatin	OATPs, Pgp, MRP2, BCRP	11.31	0.80	0.92	9.41	0.38	12.80	0,.9	0.52	0.81	1.13
Pravastatin	OATPs, MRP2	7.61	1.14	1.50	0.90	0.19	0.80	0.58	2.40	0.30	1.09
Atorvastatin	OATPs, MCT, Pgp, MRP2, BCRP	3.93	1.44	7.53	1.28	2.98	4.34	2.36	1.09	1.66	5.76

*BCRP, breast cancer resistance protein; GLUT1, glucose transporters; LAT1, large neutral amino acid transporter; MCT, monocarboxylic acid transporter; MRP, multidrug resistance-associated proteins; OAT, organic anion transporter; OATP, organic anion-transporting polypeptide; PDR, permeability directional ratio; Pgp, P glycoprotein*.

**Figure 11:**
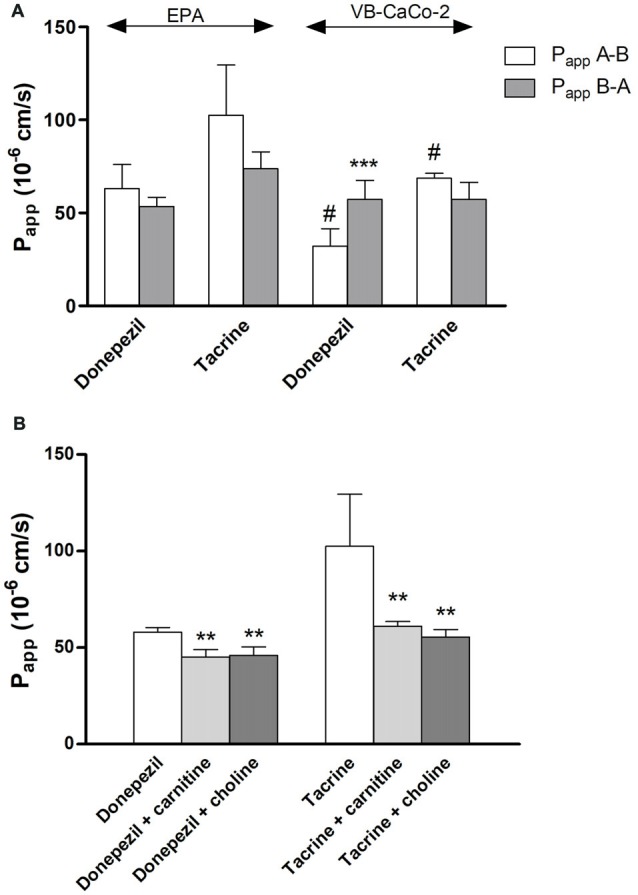
Permeability coefficients (P_app_) for donepezil and tacrine on primary rat brain endothelial cell-based BBB model (EPA) and epithelial cell line VB-Caco-2 **(A)**. Transport of donepezil and tacrine in the presence of choline (50 μM) and L-carnitine (50 μM) on the EPA model **(B)**. Statistics: mean ± SD, *n* = 4, two-way ANOVA and Bonferroni test; **P < 0.01; ***P < 0.001 compared to control; ^#^*P* < 0.05 compared to EPA.

We tested the penetration of two additional SLC ligands, the organic cation tacrine and donepezil on the EPA and VB-Caco-2 models (Figure 11A). The P_app_ of these two anticholinergic CNS drugs were high among the tested SLC related drugs in the EPA model (donepezil: 63 ± 13 10^–6^ cm/s; tacrine: 102 ± 27 10^–6^ cm/s). In primary brain endothelial cells significantly higher P_app_ values were measured for both drugs compared to those of the epithelial cells. The permeability of donepezil was higher in the A-B direction (blood-to-brain) in the BBB model indicating preferential influx transport. In the epithelial model the donepezil permeability was higher in the opposite, B-A (brain-to-blood) direction as reflected in their PDR values (0.85 in EPA vs. 1.78 in VB-Caco-2). In the BBB model the penetration of both drugs could be inhibited significantly with the endogenous cationic metabolites/nutrients choline and carnitine (Figure 11B), indicating that the drugs and the endogenous ligands may share common transporter(s).

## Discussion

A large number of BBB culture models are used in basic as well as applied research and detailed characterization and comparative datasets are needed to select the appropriate model for a particular research aim. However, such studies are scarce. The present work on nine different primary cell and cell line-based models is unique, no such comparative study with gene expression data, paracellular tightness and drug transport has been published previously.

### Comparison of EPA BBB Model to Epithelial and Brain Endothelial Cell Lines: TJ Pattern and Paracellular Barrier Tightness

**Figure 12:**
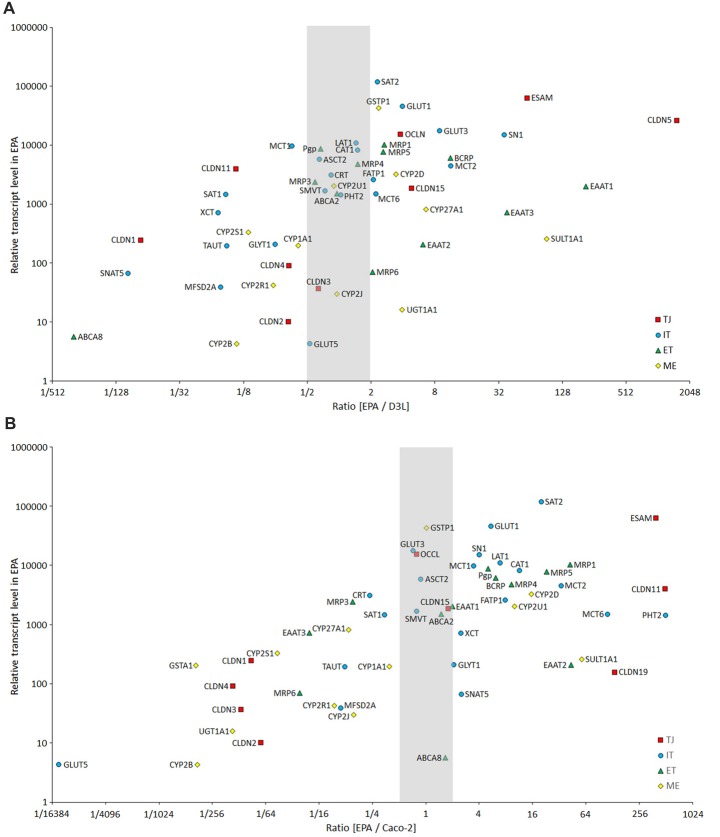
Comparison of the expression of selected BBB genes between the primary rat brain endothelial cell-based EPA model and the human brain endothelial cell line D3 **(A)**, and the human intestinal cell line Caco-2 **(B)** measured by inventoried TaqMan Gene Expression Assays. The figure shows the ratio between the gene expression level of tight junction proteins (TJ, red square), influx transporters (IT, blue dot), efflux transporters (ET, green triangle) and selected metabolic enzymes (ME, yellow diamond) in EPA and D3 or Caco-2 cells. The graph also displays the expression values in the EPA sample on the y-axis to demonstrate the level of expression of studied genes.

Our results confirm that the mRNA pattern of TJ proteins in the primary culture-based EPA BBB model differs from the pattern seen in epithelial cells (Figure 12). In the Caco-2 and MDCK cells the highest expression was measured for CLDN1, CLDN3, CLDN4 and CLDN7 genes, which is a typical pattern for intestinal epithelium *in vivo* (Chiba et al., 2008). In contrast to Caco-2 epithelial cells and in agreement with brain capillary data CLDN19, which has a tightening potential could be detected in the rat BBB model (Ohtsuki et al., 2008). Despite the different TJ transcript patterns, the TEER of epithelial cells, except for MDCK cells, was well above the critical threshold value of 150–200 Ω cm^2^, signifying a paracellular integrity necessary for permeability assays (Gaillard and de Boer, 2000; Deli et al., 2005). The reason for the low TEER value of the kidney epithelial cells is the high expression of the genes encoding the pore forming CLDN2, CLDN7 and CLDN16 which facilitate cation permeability, thereby decrease TEER (Krause et al., 2008). In spite of the low TEER values, MDCK cells form a tight paracellular barrier for small marker molecules of passive permeability (Veszelka et al., 2011; Hellinger et al., 2012).

In agreement with literature data on gene expression of rodent brain microvessels (Enerson and Drewes, 2005; Ohtsuki et al., 2008) and according to our expectations the endothelial specific ESAM and CLDN5 were measured in the EPA at the highest expression level. ESAM was described as a cell surface protein present at interendothelial cell contacts (Nasdala et al., 2002). It is expressed in all vascular endothelium. ESAM immunostaining was localized to the cell border in bEnd.3 mouse brain endothelial cell line, and co-localized with TJ proteins claudin-5 and occludin in mouse brain capillaries by electron microscopy (Nasdala et al., 2002). While claudin-5 is known to tighten BBB, no functional data are known on how ESAM contributes to brain endothelial barrier tightness.

The only transmembrane TJ protein that was unequivocally proven to contribute to the restriction of hydrophilic small molecule permeability at the BBB *in vivo* is claudin-5 (Nitta et al., 2003), this was the reason why we selected CLDN5 protein for immunostaining. CLDN5 immunostaining was well visible on the cell border of brain endothelial cells in co-culture indicating junctional tightness verified by TEER and permeability measurements. The low expression level of the CLDN5 and occludin genes measured in all the immortalized brain endothelial cell lines is responsible for the weak paracellular barrier properties, the low TEER and the high permeability values. In accordance with the gene expression data, the CLDN5 immunostaining was very weak or undetectable in these cells. Since these cell lines do not form a restrictive paracellular barrier, they are not suitable for screening small molecular drug candidates (Veszelka et al., 2011).

In the case of brain endothelial cell lines, culture media that were originally described for them were used. The culture medium of the D3 cell line contained more supplements than the medium of the primary brain endothelial cells. Despite this complex medium D3 cells still did not form a tight barrier, indicating that the cells’ intrinsic properties are responsible for the weak junctions. To improve barrier properties, brain endothelial cell lines have already been examined in co-culture conditions. Co-culture data on the rat cell lines, RBE4 and GP8, were summarized in our previous review (Deli et al., 2005). In RBE4 cells the permeability of the sucrose marker molecule was in all conditions higher than the accepted level for small molecule testing (Deli et al., 2005). Co-culture of GP8 rat brain endothelial cells with C6 glioma still resulted in very low TEER and high fluorescein permeability values (Deli et al., 2005). These data indicate, that the weak barrier properties of these two rat cell lines were not significantly improved by astrocytic influence. The basic permeability of D3 monolayers is about 10 times higher for small molecule tracers than in primary BBB models (Helms et al., 2016), similarly to our findings. Co-culture of D3 cells with both human astrocytes and pericytes did not elevate TEER as compared to mono-cultures (Hatherell et al., 2011; Helms et al., 2016). Taken together, these literature data indicate, that cell culture supplements and co-culture conditions do not improve the barrier tightness of the examined brain endothelial cell lines to such an extent that they would be suitable for drug penetration screening.

### Comparison of EPA BBB Model to Epithelial and Brain Endothelial Cell Lines: Efflux Transporters

Pgp and BCRP were identified as the two primary efflux transporters at the BBB in both human (Shawahna et al., 2011; Uchida et al., 2011) and rat brain microvessels (Enerson and Drewes, 2005; Hoshi et al., 2013). The rat EPA BBB model expressed a similarly high amount of mRNA for Pgp and BCRP. In native Caco-2 cells the expression of Pgp and BCRP genes was lower than in the EPA model (Figure 12). Vinblastine treatment significantly elevated the Pgp gene expression in VB-Caco-2 cells in agreement with the literature data (Hellinger et al., 2010). MDCK-MDR1 cells also expressed a higher level of the ABCB1 gene that can contribute to the high efflux potential of this cell line in agreement with our previous results (Hellinger et al., 2012). In the MDCK and MDCK-MDR1 cell lines the mRNA levels of ABCB1 determined by RT-qPCR and protein levels of Pgp determined by Western blot correlated very well (Gartzke and Fricker, 2014), suggesting that for this important BBB efflux pump mRNA levels may reflect well protein levels. We have verified in our previous work that Pgp protein is present in the rat EPA BBB model, and in the VB-Caco-2 and MDCK-MDR1 cells (Hellinger et al., 2012). In addition to Pgp, MRP1 was demonstrated at protein level by both Western blot and immunohistochemistry in our EPA model (Nakagawa et al., 2009). The canine kidney cells did not express BCRP, as it was demonstrated in an independent study (Quan et al., 2012), which should be taken into account for drug efflux studies. In the epithelial cell lines, the most dominant efflux transporters were MRP2 and MRP3, while in the EPA model MRP1 and MRP5. The data on epithelial cells are in concordance with the findings of Hayeshi et al. (2008) and Quan et al. (2012).

In RBE4 cells the Pgp mRNA expression was the lowest among brain endothelial cell lines, the BCRP gene expression was below the detection limit, and the transcript level of other ABC transporters was also low. In GP8 cells also lower Pgp mRNA level was measured, while BCRP and MRP6 genes were not expressed. Based on these data the usage of rat RBE4 or GP8 cell lines for (efflux) drug screening is not suggested. We measured a high gene expression for Pgp in the human D3 cell line and they also expressed BCRP. In D3 cell plasma membrane fractions high protein expression for Pgp, MRP1 and MRP4 were measured by LC-MS/MS-based protein quantification analysis (Ohtsuki et al., 2013), supporting our present mRNA findings.

EAAT transporters participate in the efflux transport of glutamate across the BBB and are responsible for the low level of glutamate in the brain interstitial fluid (Helms et al., 2017). L-glutamate is taken up via EAAT1 at the abluminal membrane of brain endothelial cells and exits at the luminal membrane via a low affinity glutamate/aspartate transporter. Among EAAT transporter genes, the EAAT1 gene was expressed at the highest level in brain endothelial cells while in Caco-2 and MDCK cells the expression level of EAAT3 was the highest. The mRNA level of the EAAT2 was very low in Caco-2 cells and it was not expressed in MDCK cells. In contrast to the EPA BBB model, the expression of EAAT genes was negligible in brain endothelial cell lines except for D3 cells in which the EAAT3 gene was expressed at a low level. Our results support the findings of Helms et al. (2012). They demonstrated the presence of EAAT1, -2 and -3 mRNA in brain endothelial cells by conventional RT-PCR and the localization of EAAT1 and -3 in endothelial cells by immunostaining.

### Comparison of the EPA BBB Model to Epithelial and Brain Endothelial Cell Lines: Metabolic Enzymes

Endothelial cells of brain capillaries express enzymes that are capable of modifying drugs and xenobiotics that could bypass the BBB and thereby protect the CNS from the potential harmful effects of these molecules (Deli, 2011). Specific phase I and phase II enzymes participate in the formation of this metabolic barrier, with a supposed role in local drug metabolism and transport. Our data confirm that cultured brain endothelial cells express genes for phase-I and phase-II drug-metabolizing enzymes in levels comparable to epithelial cells (Figure 12). Among the cytochrome P450 enzymes, the CYP2D6 and the CYP2U1 mRNA levels were the highest in the EPA BBB model. CYP2D6 is involved in the hepatic metabolism of many clinically used drugs, while CYP2U1 is an extrahepatic isoform expressed in the thymus and brain, which metabolize arachidonic acid and other long chain fatty acids (Dauchy et al., 2009). In Caco-2 cells, the gene of extrahepatic CYP2S1 enzyme, which metabolizes naphthalene, was expressed at the highest level. Only two enzyme genes, CYP1A2 and CYP2E1 could be tested in MDCK canine cells, which were not expressed. Gene probes for other CYP enzymes in this species were unavailable. In D3 cells the CYP2S1 and the CYP2U1 genes were most dominantly expressed, in concordance with the data of Dauchy et al. (2009). In GP8 and RBE4 cell lines all the genes of the tested phase-I enzymes were expressed at very low or negligible levels except for the CYP1A1 gene in RBE4 cells, which encodes an enzyme metabolizing arachidonic acid and other polyunsaturated fatty acids into signaling molecules. We found in a previous work that the arachidonic acid metabolism and the vasoactive properties of GP8 and RBE4 cell lines are altered compared to primary brain endothelial cells (Kis et al., 1999).

From the tested phase-II metabolic enzymes involved in the cellular detoxification processes, the glutathione S-transferase π (GSTP1) gene was expressed at the highest level in the EPA model. GSTP1 is expressed in brain capillaries, where it colocalizes to a large extent with MRP2 at the luminal plasma membrane of brain endothelial cells (Bauer et al., 2008). Colocalization and coordinated upregulation of MRP2 and GSTP1 by pregnane X receptor activation suggest functional coupling of this metabolizing enzyme and efflux transporter (Bauer et al., 2008). In Caco-2 cells, the genes of tested phase-II metabolic enzymes were transcribed at high levels, except SULT1A1. GSTP1 gene expression was high in brain endothelial cell lines. GSTA1, SULT1A1 and UGT1A1 genes were expressed in the EPA, but were absent or detected at very low level in brain endothelial cell lines. Summarizing these data, phase-I and phase-II metabolic enzymes are expressed in brain endothelial cells of the EPA model, suggesting a role in the regulation of local drug transport.

### Comparison of the EPA BBB Model to Epithelial and Brain Endothelial Cell Lines: Influx Transporters and Drug Permeability

In the present study, we compared the expression levels of 25 influx and 11 efflux transporter genes in nine different culture models. To check the functionality of these transporters we tested nine different drugs on EPA, Caco-2 and MDCK cells, which showed the best paracellular tightness properties among the models. We excluded the brain endothelial cell lines, because they did not form a restrictive paracellular barrier to screening the permeability of small molecules.

The dominant SLC transporter for hexoses at the mammalian BBB is the GLUT1, which provides D-glucose, the primary source of energy for brain functions (Shawahna et al., 2011). The EPA model, expressed the highest level of the GLUT1 gene of all the models. We have previously verified the presence of GLUT1 protein by both Western blot and immunohistochemistry in the EPA model (Nakagawa et al., 2009). GLUT3 mRNA was also present in this BBB model, but at a lower level, in concordance with rat and human brain microvessel gene and protein data (Enerson and Drewes, 2005; Shawahna et al., 2011; Uchida et al., 2011; Hoshi et al., 2013). In contrast to BBB data, we found that in Caco-2 cells GLUT3 and GLUT5 are the dominant hexose transporters, not GLUT1. Similar mRNA data were obtained on Caco-2 cells by other groups (Hayeshi et al., 2008). SLC transporter genes, that were expressed at a high mRNA level in our study, like GLUT-1, MCT-1, LAT-1 and PEPT1 were all demonstrated in Caco-2 cells by proteomic analysis, too (Ölander et al., 2016). Moreover, a correlation was found between normalized mRNA rank and normalized protein abundance rank in Caco-2 cells for selected SLC genes (Ölander et al., 2016). The renal MDCK cell line expressed GLUT1 at high level, as it was already published (Quan et al., 2012), but not the other two GLUT transporters.

Similarly to the primary BBB model, the dominant glucose transporter in D3 cells was also GLUT1, in agreement with literature data (Carl et al., 2010; Urich et al., 2012; Ohtsuki et al., 2013). In GP8 cells the main glucose transporter was also GLUT1, while in RBE4 cells it was GLUT3. In RBE4, as well as in primary rat brain endothelial cells, both the 55 kDa GLUT1 and a 45–50 kDa band corresponding to brain GLUT3 were detected by Western blot analysis (Régina et al., 2001), in concordance with our mRNA findings. The expression level of the GLUT5 gene was low or not detectable in the endothelial cell lines. We tested GLUT1 functionality on the EPA model, and using glucopyranose as a transporter ligand (Bidder, 1968) we found a higher P_app_ value as compared to the epithelial cell lines and a very low PDR value, suggesting influx transport. These data are the first functional results on GLUT1 in a rat BBB culture model. The functionality of GLUT1 was only proved on bovine and human stem cell-based BBB culture models so far (Helms et al., 2016).

The MCT transporter family provides the CNS with the secondary energy source ketone bodies, like lactate, and also with thyroid hormones. Lactate is used by the human brain during development and the postnatal period, and in adult life during starvation, diabetes and ischemic insults to maintain energy homeostasis in the CNS (Campos-Bedolla et al., 2014). Lactate is bidirectionally transported by MCTs, among which the principal transporter at the BBB is MCT1 both in rodents and humans (Enerson and Drewes, 2005; Dahlin et al., 2009; Shawahna et al., 2011). The EPA model expressed high levels of all three tested MCT genes. Caco-2 cells expressed MCT1 at a lower level, and much less MCT8 and -6. MDCK cells did not express MCT1. In contrast, all brain endothelial cells except RBE4 expressed this transporter well. Besides monocarboxylates, MCTs participate also in the transport of drugs like salicylic acid or probenecid at the BBB (Enerson and Drewes, 2003; Bhattacharya and Boje, 2006). The functional presence of the MCT transporters at the EPA model was proved by the moderate permeability rate of probenecid, an organic anion, and a ligand for MCTs and organic anion transporter systems (OAT, SLC22; Deguchi et al., 2000). The low permeability of salicylic acid, a substrate for MCT1, OATP2 and MRP4 at the EPA model might be explained by the potential counteracting vectorial transport of SLC transporters and MRP efflux pumps. Significantly higher permeability was measured for probenecid on the Caco-2 cell lines, and for salicylic acid on the epithelial cells than on the BBB model, which might be explained by the different expression pattern for SLC and ABC transporters in the BBB model vs. epithelial cell lines.

All models expressed high levels of SLC transporters for amino acids, with significantly higher levels of large amino acid transporters CAT1, LAT1 and small amino acid transporters SAT2 and SN1 in the EPA model compared to epithelial models. The expression of LAT1 gene is the highest SLC in human brain microvessels (Shawahna et al., 2011), and is approximately 100-fold greater than in other tissues. LAT1, the most abundant amino acid carrier, is selectively expressed on both plasma membranes of brain capillaries. LAT1 supplies leucine, tryptophan, tyrosine and phenylalanine to the brain and participates in the transport of drugs like L-DOPA, baclofen, valproic acid and gabapentin across the BBB (Ohtsuki and Terasaki, 2007). L-DOPA, a well-known example of LAT1 substrates, gave the highest P_app_ value among all tested drugs in the BBB model. For valproic acid, which is also transported by OATPs (Taogoshi et al., 2005), higher P_app_ values were measured in the Caco-2 and lower in the MDCK cell lines than in the EPA model. In the BBB model, baclofen had significantly higher P_app_ compared to the two epithelial cell lines. Similar results were obtained for baclofen on bovine brain endothelial cells vs. epithelial culture models (Hakkarainen et al., 2010). Among the LAT1 transported drugs, gabapentin, which has a significant efflux transport by ABC transporters (Nakanishi et al., 2013), had the lowest permeability on all five models. The transfer of gabapentin across the VB-Caco-2 cell line was below the detection limit, in accordance with the strong efflux properties of this cell line (Hellinger et al., 2012).

The gene expression of the tested two peptide transporters was strikingly different among the models. A high level of PEPT1 mRNA in the Caco-2 models and no expression in EPA and MDCK models, in contrast a high transcript level of PHT2 in the EPA model and no expression in Caco-2 models were measured, suggesting that peptide transport must be very different on these models. PHT2 was identified as a BBB-related SLC transporter in two independent studies (Enerson and Drewes, 2005; Dahlin et al., 2009), confirming our results. MFSD2A transports DHA in the form of lysophosphatidylcholine in a sodium-dependent manner. MFSD2A is selectively expressed in brain capillaries and mediate the brain uptake of DHA. The brain endothelial expression of MFSD2A is regulated by pericytes *in vivo* (Ben-Zvi et al., 2014). This is the first study to compare the gene expression level of this important BBB transporter in nine different models. The MFSD2A gene was expressed in all models, except in GP8 and RBE4 cell lines, and showed a higher mRNA expression in the epithelial models and D3 cells than in the EPA model. All models expressed high to moderate levels of mRNA for SLC transporters of fatty acids, glycine, taurine, creatinine and vitamin C. There was no significant difference in SLC transporter gene expression between Caco-2 and VB-Caco-2 cells, except down-regulation for MCT8. In the D3 cell line the highest expression among the SLC transporters was for SAT2, followed by GLUT1, LAT1 and MCT1 in our study, as well as in the literature (Carl et al., 2010; Urich et al., 2012). Lithium treatment of D3 cells, which increase BBB properties by the Wnt pathway (Weksler et al., 2013), upregulated the expression level in half of the tested carrier and transporter genes, such as GLUT3, -5, MCT8, SN1, SNAT5, PEPT1, FATP1, ABCA2, GLYT1, TAUT, CRT, ASCT2.

Exogenous substrates of OATPs include antibiotics, antidiabetic and anti-inflammatory drugs, antivirals, antihistamines, antihypertensives, immunosuppressants, and anticancer drugs, thus OATPs at the BBB are important regulators of CNS drug disposition (Campos-Bedolla et al., 2014). Statins were also identified as substrates of OATPs (Kalliokoski and Niemi, 2009). There is an increasing interest in statins to use them in neuronal diseases, such as stroke, Parkinson’s disease, or Alzheimer’s disease (Malfitano et al., 2014), but brain penetration is the key for their potential therapeutic efficacy. In the present study, the permeability of rosuvastatin, pravastatin and atorvastatin was compared on five models. The higher penetration of the tested three statins across the EPA model as compared to epithelial cells may be explained by the higher expression of Oatp-1a2 and -1c1 influx transporters in brain endothelial cells, and the stronger efflux mechanisms, especially MRP2 in epithelial cells. In agreement with our observation, very low apical to basal P_app_ values were measured for rosuvastatin and atorvastatin in Caco-2 cells (Li et al., 2011). In this article, the role of Pgp, BCRP, and MRP2 in the efflux transport of these statin was also proven.

Tacrine and donepezil are two anticholinergic drugs with good brain penetration, approved for the treatment of Alzheimer’s disease. Using an immortalized brain endothelial cell line, the organic cation transporter-2 (OCT2, SLC22A2), the organic cation/carnitine transporter OCTN2 (SLC22A5), and the choline transporter CHT1 (SLC5A7) were identified as influx transporters of these drugs (Kang et al., 2005; Lee et al., 2012). We also found a high permeability for both drugs on the EPA BBB model. The endogenous cationic metabolites choline and carnitine could significantly inhibit the penetration of tacrine and donepezil, indicating that the drugs and the endogenous ligands may share common transporters. Lower permeability was measured for these two drugs on VB-Caco-2 cells, and for donepezil the basal to apical permeability was higher, in contrast to the EPA model.

In conclusion, our study reveals major differences in the gene expression patterns between the primary cell-based BBB model and epithelial or brain endothelial cell lines for several key BBB related genes. Epithelial cell line models showed appropriate paracellular tightness, even if the pattern for TJ protein genes were distinct between epithelial cell lines and the BBB model. Disparity in the gene expression of transporters between BBB and epithelial models were also reflected in the permeability of selected drugs. These findings emphasize the growing importance of SLC-mediated drug targeting to brain and the use of appropriate culture models. Among the tested culture models, the primary cell-based EPA model is suitable for the functional analysis of the BBB.

## Author Contributions

MD, SV, MV and GR: conceived and designed the experiments. SV, AT, FW, AT, IG, MM, AB and ÉH: performed the experiments. SV, AT, MV and MD: analyzed the data. MD, MV and GR: contributed reagents/materials/analysis tools. SV, AT, FW, AT, MV, GR and MD: wrote and edited the article.

## Conflict of Interest Statement

The authors ÉH and MV are employed by the company Gedeon Richter Plc. The other authors declare that the research was conducted in the absence of any commercial or financial relationships that could be construed as a potential conflict of interest.

## References

[B1] Abbott N. J. (2013). Blood-brain barrier structure and function and the challenges for CNS drug delivery. J. Inherit. Metab. Dis. 36, 437–449. 10.1007/s10545-013-9608-0 23609350

[B2] Artursson P. Palm K. Luthman K. (2001). Caco-2 monolayers in experimental and theoretical predictions of drug transport. Adv. Drug Deliv. Rev. 46, 27–43. 10.1016/S0169-409X(00)00128-9 11259831

[B3] Avdeef A. Deli M. A. Neuhaus W. (2015). “*In vitro* assays for assessing BBB permeability: artificial membrane and cell culture models,” in Blood-Brain Barrier in Drug Discovery: Optimizing Brain Exposure of CNS Drugs and Minimizing Brain Side Effects for Peripheral Drugs, eds Di L. Kerns E. H. (New Jersey, NJ: John Wiley & Sons), 188–237.

[B4] Banks W. A. (2016). From blood-brain barrier to blood-brain interface: new opportunities for CNS drug delivery. Nat. Rev. Drug Discov. 15, 275–292. 10.1038/nrd.2015.21 26794270

[B5] Bauer B. Hartz A. M. Lucking J. R. Yang X. Pollack G. M. Miller D. S. (2008). Coordinated nuclear receptor regulation of the efflux transporter, Mrp2, and the phase-II metabolizing enzyme, GSTpi, at the blood-brain barrier. J. Cereb. Blood Flow Metab. 28, 1222–1234. 10.1038/jcbfm.2008.16 18349876

[B6] Ben-Zvi A. Lacoste B. Kur E. Andreone B. J. Mayshar Y. Yan H.. (2014). Mfsd2a is critical for the formation of the blood-brain barrier. Nature 509, 507–511. 10.1038/nature13324 24828040 PMC4134871

[B7] Bhattacharya I. Boje K. M. (2006). Potential gamma-hydroxybutyric acid (GHB) drug interactions through blood-brain barrier transport inhibition: a pharmacokinetic simulation-based evaluation. J. Pharmacokinet. Pharmacodyn. 33, 657–681. 10.1007/s10928-006-9029-x 16941233

[B8] Bidder T. G. (1968). Hexose translocation across the blood-brain interface: configurational aspects. J. Neurochem. 15, 867–874. 10.1111/j.1471-4159.1968.tb10333.x 18561500

[B9] Booth R. Kim H. (2014). Permeability analysis of neuroactive drugs through adynamic microfluidic *in vitro* blood-brain barrier model. Ann. Biomed. Eng. 42, 2379–2391. 10.1007/s10439-014-1086-5 25118670

[B10] Campos-Bedolla P. Walter F. R. Veszelka S. Deli M. A. (2014). Role of the blood-brain barrier in the nutrition of the central nervous system. Arch. Med. Res. 45, 610–638. 10.1016/j.arcmed.2014.11.018 25481827

[B11] Carl S. M. Lindley D. J. Das D. Couraud P. O. Weksler B. B. Romero I.. (2010). ABC and SLC transporter expression and proton oligopeptide transporter (POT) mediated permeation across the human blood—brain barrier cell line, hCMEC/D3. Mol. Pharm. 7, 1057–1068. 10.1021/mp900178j 20524699 PMC2914114

[B12] Chiba H. Osanai M. Murata M. Kojima T. Sawada N. (2008). Transmembrane proteins of tight junctions. Biochim. Biophys. Acta 1778, 588–600. 10.1016/j.bbamem.2007.08.017 17916321

[B14] Cucullo L. Hossain M. Puvenna V. Marchi N. Janigro D. (2011). The role of shear stress in blood-brain barrier endothelial physiology. BMC Neurosci. 12:40. 10.1186/1471-2202-12-40 21569296 PMC3103473

[B15] Cucullo L. Hossain M. Tierney W. Janigro D. (2013). A new dynamic *in vitro* modularcapillaries-venules modular system: cerebrovascular physiology in a box. BMC Neurosci. 14:18. 10.1186/1471-2202-14-18 23388041 PMC3598202

[B16] Dahlin A. Royall J. Hohmann J. G. Wang J. (2009). Expression profiling of the solute carrier gene family in the mouse brain. J. Pharmacol. Exp. Ther. 329, 558–570. 10.1124/jpet.108.149831 19179540 PMC2672879

[B17] Dauchy S. Miller F. Couraud P. O. Weaver R. J. Weksler B. Romero I. A.. (2009). Expression and transcriptional regulation of ABC transporters and cytochromes P450 in hCMEC/D3 human cerebral microvascular endothelial cells. Biochem. Pharmacol. 77, 897–909. 10.1016/j.bcp.2008.11.001 19041851

[B18] Deguchi Y. Yokoyama Y. Sakamoto T. Hayashi H. Naito T. Yamada S.. (2000). Brain distribution of 6-mercaptopurine is regulated by the efflux transport system in the blood-brain barrier. Life Sci. 66, 649–662. 10.1016/s0024-3205(99)00637-2 10794520

[B19] Dehouck M. P. Méresse S. Delorme P. Fruchart J. C. Cecchelli R. (1990). An easier, reproducible and mass-production method to study the blood-brain barrier *in vitro*. J. Neurochem. 54, 1798–1801. 10.1111/j.1471-4159.1990.tb01236.x 2182777

[B20] Deli M. A. (2011). “Drug transport and the blood-brain barrier,” in Solubility, Delivery, and ADME Problems of Drugs and Drug-Candidates, eds Tihanyi K. Vastag M. (Washington, DC: Bentham Science Publ. Ltd.), 144–165.

[B21] Deli M. A. Abrahám C. S. Kataoka Y. Niwa M. (2005). Permeability studies on *in vitro* blood-brain barrier models: physiology, pathology, and pharmacology. Cell. Mol. Neurobiol. 25, 59–127. 10.1007/s10571-004-1377-8 15962509 PMC11529645

[B23] Enerson B. E. Drewes L. R. (2003). Molecular features, regulation, and function of monocarboxylate transporters: implications for drug delivery. J. Pharm. Sci. 92, 1531–1544. 10.1002/jps.10389 12884241

[B22] Enerson B. E. Drewes L. R. (2005). The rat blood—brain barrier transcriptome. J. Cereb. Blood Flow Metab. 26, 959–973. 10.1038/sj.jcbfm.9600249 16306934

[B24] Evers R. Kool M. Smith A. J. van Deemter L. de Haas M. Borst P. (2000). Inhibitory effect of the reversal agents V-104, GF120918 and Pluronic L61 on MDR1 Pgp-, MRP1- and MRP2-mediated transport. Br. J. Cancer 83, 366–374. 10.1054/bjoc.2000.1260 10917553 PMC2374556

[B26] Gaillard P. J. de Boer A. G. (2000). Relationship between permeability status of the blood-brain barrier and *in vitro* permeability coefficient of a drug. Eur. J. Pharm. Sci. 12, 95–102. 10.1016/s0928-0987(00)00152-4 11102736

[B27] Garberg P. Bal L. M. Borg N. Cecchelli R. Fenart L. Hurst R. D.. (2005). *In vitro* models of the blood-brain barrier. Toxicol. in vitro 19, 299–334. 10.1016/j.tiv.2004.06.011 15713540

[B28] Gartzke D. Fricker G. (2014). Establishment of optimized MDCK cell lines for reliable efflux transport studies. J. Pharm. Sci. 103, 1298–1304. 10.1002/jps.23901 24532159

[B29] Greenwood J. Pryce G. Devine L. Male D. K. dos Santos W. L. Calder V. L.. (1996). SV40 large T immortalised cell lines of the rat blood-brain and blood-retinal barriers retain their phenotypic and immunological characteristics. J. Neuroimmunol. 71, 51–63. 10.1016/s0165-5728(96)00130-0 8982103

[B30] Griep L. Wolbers F. de Wagenaar B. ter Braak P. Weksler B. Romero I. A.. (2013). BBB on chip: microfluidic platform to mechanically and biochemically modulate blood-brain barrier function. Biomed. Microdevices 15, 145–150. 10.1007/s10544-012-9699-7 22955726

[B31] Hakkarainen J. J. Jalkanen A. J. Kääriäinen T. M. Keski-Rahkonen P. Venäläinen T. Hokkanen J.. (2010). Comparison of *in vitro* cell models in predicting *in vivo* brain entry of drugs. Int. J. Pharm. 402, 27–36. 10.1016/j.ijpharm.2010.09.016 20920560

[B32] Hatherell K. Couraud P. O. Romero I. A. Weksler B. Pilkington G. J. (2011). Development of a three-dimensional, all-human *in vitro* model of the blood-brain barrier using mono-, co-, and tri-cultivation Transwell models. J. Neurosci. Methods 199, 223–229. 10.1016/j.jneumeth.2011.05.012 21609734

[B501] Hayeshi R. Hilgendorf C. Artursson P. Augustijns P. Brodin B. Dehertogh P.. (2008). Comparison of drug transporter gene expression and functionality in Caco-2 cells from 10 different laboratories. Eur. J. Pharm. Sci. 35, 383–396. 10.1016/j.ejps.2008.08.004 18782614

[B33] Hellinger É Bakk M. L. Pócza P. Tihanyi K. Vastag M. (2010). Drug penetration model of vinblastine-treated Caco-2 cultures. Eur. J. Pharm. Sci. 41, 96–106. 10.1016/j.ejps.2010.05.015 20595016

[B607] Hellinger É Veszelka S. Tóth A. E. Walter F. Kittel A. Bakk M. L.. (2012). Comparison of brain capillary endothelial cell-based and epithelial (MDCK-MDR1, Caco-2, and VB-Caco-2) cell-based surrogate blood-brain barrier penetration models. Eur. J. Pharm. Biopharm. 82, 340–351. 10.1016/j.ejpb.2012.07.020 22906709

[B34] Helms H. C. Abbott N. J. Burek M. Cecchelli R. Couraud P. O. Deli M. A.. (2016). *In vitro* models of the blood-brain barrier: an overview of commonly used brain endothelial cell culture models and guidelines for their use. J. Cereb. Blood Flow Metab. 36, 862–890. 10.1177/0271678x16630991 26868179 PMC4853841

[B35] Helms H. C. Madelung R. Waagepetersen H. S. Nielsen C. U. Brodin B. (2012). *In vitro* evidence for the brain glutamate efflux hypothesis: brain endothelial cells cocultured with astrocytes display a polarized brain-to-blood transport of glutamate. Glia 60, 882–893. 10.1002/glia.22321 22392649

[B36] Helms H. C. C. Nielsen C. U. Waagepetersen H. S. Brodin B. (2017). Glutamate transporters in the blood-brain barrier. Adv. Neurobiol. 16, 297–314. 10.1007/978-3-319-55769-4_15 28828617

[B37] Hoheisel D. Nitz T. Franke H. Wegener J. Hakvoort A. Tilling T.. (1998). Hydrocortisone reinforces the blood-brain barrier properties in a serum free cell culture system. Biochem. Biophys. Res. Commun. 247, 312–315. 10.1006/bbrc.1997.8051 9679029

[B502] Hoshi Y. Uchida Y. Tachikawa M. Inoue T. Ohtsuki S. Terasaki T. (2013). Quantitative atlas of blood-brain barrier transporters, receptors, and tight junction proteins in rats and common marmoset. J. Pharm. Sci. 102, 3343–3355. 10.1002/jps.23575 23650139

[B38] Kalliokoski A. Niemi M. (2009). Impact of OATP transporters on pharmacokinetics. Br. J. Pharmacol. 158, 693–705. 10.1111/j.1476-5381.2009.00430.x 19785645 PMC2765590

[B39] Kang Y. S. Lee K. E. Lee N. Y. Terasaki T. (2005). Donepezil, tacrine and a-phenyl-ntert- butyl nitrone (PBN) inhibit choline transport by conditionally immortalized rat brain capillary endothelial cell lines (TR-BBB). Arch. Pharm. Res. 28, 443–450. 10.1007/bf02977674 15918518

[B40] Kis B. Szabó C. A. Pataricza J. Krizbai I. A. Mezei Z. Gecse A.. (1999). Vasoactive substances produced by cultured rat brain endothelial cells. Eur. J. Pharmacol. 368, 35–42. 10.1016/s0014-2999(99)00024-2 10096767

[B41] Krause G. Winkler L. Mueller S. L. Haseloff R. F. Piontek J. Blasig I. E. (2008). Structure and function of claudins. Biochim. Biophys. Acta 1778, 631–645. 10.1016/j.bbamem.2007.10.018 18036336

[B42] Kürti L. Veszelka S. Bocsik A. Dung N. T. Ozsvári B. Puskás L. G.. (2012). The effect of sucrose esters on a culture model of the nasal barrier. Toxicol. in vitro 26, 445–454. 10.1016/j.tiv.2012.01.015 22274662

[B43] Lee N. Y. Choi H. O. Kang Y. S. (2012). The acetylcholinesterase inhibitors competitively inhibited an acetyl L-carnitine transport through the blood-brain barrier. Neurochem. Res. 37, 1499–1507. 10.1007/s11064-012-0723-3 22359054

[B503] Li J. Volpe D. A. Wang Y. Zhang W. Bode C. Owen A.. (2011). Use of transporter knockdown Caco-2 cells to investigate the *in vitro* efflux of statin drugs. Drug Metab. Dispos. 39, 1196–1202. 10.1124/dmd.111.038075 21447733

[B44] Livak K. J. Schmittgen T. D. (2001). Analysis of relative gene expression data using real-time quantitative PCR and the 2^−ΔΔ*C*_T_^ method. Methods 25, 402–408. 10.1006/meth.2001.1262 11846609

[B45] Malfitano A. M. Marasco G. Proto M. C. Laezza C. Gazzerro P. Bifulco M. (2014). Statins in neurological disorders: an overview and update. Pharmacol. Res. 88, 74–83. 10.1016/j.phrs.2014.06.007 24954580

[B46] Nakagawa S. Deli M. A. Kawaguchi H. Shimizudani T. Shimono T. Kittel A.. (2009). A new blood-brain barrier model using primary rat brain endothelial cells, pericytes and astrocytes. Neurochem. Int. 54, 253–263. 10.1016/j.neuint.2008.12.002 19111869

[B47] Nakanishi H. Yonezawa A. Matsubara K. Yano I. (2013). Impact of P-glycoprotein and breast cancer resistance protein on the brain distribution of antiepileptic drugs in knockout mouse models. Eur. J. Pharmacol. 710, 20–28. 10.1016/j.ejphar.2013.03.049 23588114

[B48] Nasdala I. Wolburg-Buchholz K. Wolburg H. Kuhn A. Ebnet K. Brachtendorf G.. (2002). A transmembrane tight junction protein selectively expressed on endothelial cells and platelets. J. Biol. Chem. 277, 16294–16303. 10.1074/jbc.M111999200 11847224

[B49] Nitta T. Hata M. Gotoh S. Seo Y. Sasaki H. Hashimoto N.. (2003). Size-selective loosening of the bloodbrain barrier in claudin-5-deficient mice. J. Cell Biol. 161, 653–660. 10.1083/jcb.200302070 12743111 PMC2172943

[B504] Ohtsuki S. Ikeda C. Uchida Y. Sakamoto Y. Miller F. Glacial F.. (2013). Quantitative targeted absolute proteomic analysis of transporters, receptors and junction proteins for validation of human cerebral microvascular endothelial cell line hCMEC/D3 as a human blood-brain barrier model. Mol. Pharm. 10, 289–296. 10.1021/mp3004308 23137377

[B50] Ohtsuki S. Terasaki T. (2007). Contribution of carrier-mediated transport systems to the blood-brain barrier as a supporting and protecting interface for the brain; importance for CNS drug discovery and development. Pharm. Res. 24, 1745–1758. 10.1007/s11095-007-9374-5 17619998

[B51] Ohtsuki S. Yamaguchi H. Katsukura Y. Asashima T. Terasaki T. (2008). mRNA expression levels of tight junction protein genes in mouse brain capillary endothelial cells highly purified by magnetic cell sorting. J. Neurochem. 104, 147–154. 10.1111/j.1471-4159.2007.05008.x 17971126

[B52] Ölander M. Wiśniewski J. R. Matsson P. Lundquist P. Artursson P. (2016). The proteome of filter-grown Caco-2 cells with a focus on proteins involved in drug disposition. J. Pharm. Sci. 105, 817–827. 10.1016/j.xphs.2015.10.030 26869432

[B53] Paolinelli R. Corada M. Ferrarini L. Devraj K. Artus C. Czupalla C. J.. (2013). Wnt activation of immortalized brain endothelial cells as a tool for generating a standardized model of the blood brain barrier *in vitro*. PLoS One 8:e70233. 10.1371/journal.pone.0070233 23940549 PMC3734070

[B54] Pardridge W. M. (2015). Blood-brain barrier endogenous transporters as therapeutic targets: a new model for small molecule CNS drug discovery. Expert Opin. Ther. Targets. 19, 1059–1072. 10.1517/14728222.2015.1042364 25936389

[B55] Patabendige A. Skinner R. A. Abbott N. J. (2013). Establishment of a simplified *in vitro* porcine blood-brain barrier model with high transendothelial electrical resistance. Brain Res. 1521, 1–15. 10.1016/j.brainres.2012.06.057 22789905 PMC3694297

[B56] Perrière N. Demeuse P. Garcia E. Regina A. Debray M. Andreux J. P.. (2005). Puromycin-based purification of rat brain capillary endothelial cell cultures. Effect on the expression of blood-brain barrier-specific properties. J. Neurochem. 93, 279–289. 10.1111/j.1471-4159.2004.03020.x 15816851

[B57] Prabhakarpandian B. Shen M.-C. Nichols J. B. Mills I. R. Sidoryk-Wegrzynowicz M. Aschner M.. (2013). SyM-BBB: a microfluidic blood brain barrier model. Lab Chip 13, 1093–1101. 10.1039/c2lc41208j 23344641 PMC3613157

[B505] Quan Y. Jin Y. Faria T. N. Tilford C. A. He A. Wall D. A.. (2012). Expression profile of drug and nutrient absorption related genes in Madin-Darby canine kidney (MDCK) cells grown under differentiation conditions. Pharmaceutics 4, 314–333. 10.3390/pharmaceutics4020314 24300234 PMC3834914

[B58] Régina A. Morchoisne S. Borson N. D. McCall A. L. Drewes L. R. Roux F. (2001). Factor(s) released by glucose-deprived astrocytes enhance glucose transporter expression and activity in rat brain endothelial cells. Biochim. Biophys. Acta 1540, 233–242. 10.1016/s0167-4889(01)00133-1 11583818

[B59] Roux F. Durieu-Trautmann O. Chaverot N. Claire M. Mailly P. Bourre J. M.. (1994). Regulation of gamma-glutamyl transpeptidase and alkaline phosphatase activities in immortalized rat brain microvessel endothelial cells. J. Cell. Physiol. 159, 101–113. 10.1002/jcp.1041590114 7908023

[B60] Shawahna R. Uchida Y. Declèves X. Ohtsuki S. Yousif S. Dauchy S.. (2011). Transcriptomic and quantitative proteomic analysis of transporters and drug metabolizing enzymes in freshly isolated human brain microvessels. Mol. Pharm. 8, 1332–1341. 10.1021/mp200129p 21707071

[B61] Szabó C. A. Deli M. A. Ngo T. K. Joó F. (1997). Production of pure primary rat cerebral endothelial cell culture: a comparison of different methods. Neurobiology 5, 1–16. 9302692

[B62] Taogoshi T. Nomura A. Murakami T. Nagai J. Takano M. (2005). Transport of prostaglandin E1 across the blood-brain barrier in rats. J. Pharm. Pharmacol. 57, 61–66. 10.1211/0022357055173 15638994

[B63] Tóth A. E. Tóth A. Walter F. R. Kiss L. Veszelka S. Ózsvári B.. (2014). Compounds blocking methylglyoxal-induced protein modification and brain endothelial injury. Arch. Med. Res. 45, 753–764. 10.1016/j.arcmed.2014.10.009 25446614

[B506] Uchida Y. Ohtsuki S. Katsukura Y. Ikeda C. Suzuki T. Kamiie J.. (2011). Quantitative targeted absolute proteomics of human blood-brain barrier transporters and receptors. J. Neurochem. 117, 333–345. 10.1111/j.1471-4159.2011.07208.x 21291474

[B777] Urich E. Lazic S. E. Molnos J. Wells I. Freskgård P. O. (2012). Transcriptional profiling of human brain endothelial cells reveals key properties crucial for predictive *in vitro* blood-brain barrier models. PLoS One 7:e38149. 10.1371/journal.pone.0038149 22675443 PMC3364980

[B64] Vastag M. Keseru G. M. (2009). Current *in vitro* and *in silico* models of blood-brain barrier penetration: a practical view. Curr. Opin. Drug Discov. Devel. 12, 115–124. 19152220

[B888] Veszelka S. Kittel Á. Deli M. A. (2011). “Tools of modelling blood-brain barrier penetrability,” in Solubility, Delivery and ADME Problems of Drugs and Drug-Candidates, eds Tihanyi K. Vastag M. (Washington, DC: Bentham Science Publ. Ltd.), 166–188.

[B65] Veszelka S. Bocsik A. Walter F. Hantosi D. Deli M. A. (2015). Blood-brain-barrier coculture models to study nanoparticle penetration: focus on coculture systems. Acta Biol. Szeged. 59, 157–168. 22675443

[B66] Walter F. R. Valkai S. Kincses A. Petneházi A. Czeller T. Veszelka S. (2016). A versatile lab-on-a-chip tool for modeling biological barriers. Sens. Act. B Chem. 222, 1209–1219. 10.1016/j.snb.2015.07.110

[B67] Walter F. R. Veszelka S. Pásztói M. Péterfi Z. A. Tóth A. Rákhely G. (2015). Tesmilifene modifies brain endothelial functions and opens the blood-brain/blood-glioma barrier. J. Neurochem. 134, 1040–1054. 10.1111/jnc.13207 26112237

[B68] Weksler B. Romero I. A. Couraud P. O. (2013). The hCMEC/D3 cell line as a model of the human blood brain barrier. Fluids Barriers CNS 10:16. 10.1186/2045-8118-10-16 23531482 PMC3623852

[B69] Weksler B. B. Subileau E. A. Perrière N. Charneau P. Holloway K. Leveque M.. (2005). Blood-brain barrier-specific properties of a human adult brain endothelial cell line. FASEB J. 19, 1872–1874. 10.1096/fj.04-3458fje 16141364

