# Supplementary material for: Comparison of a Rat Primary Cell-Based Blood-Brain Barrier Model With Epithelial and Brain Endothelial Cell Lines: Gene Expression and Drug Transport
Source: Front Mol Neurosci. 2018 May 22;11:166. doi: 10.3389/fnmol.2018.00166 (PMC5972182; doi:10.3389/fnmol.2018.00166)
Supplement: Supplementary file 1 [file Presentation_1.PDF]

Supplementary material

Comparison of a rat primary cell based blood-brain barrier model with epithelial and brain endothelial cell lines: gene expression and drug transport

Szilvia Veszelka, András Tóth, Fruzsina R. Walter, Andrea E. Tóth, Ilona Gróf, Mária Mészáros, Alexandra Bocsik, Éva Hellinger, Monika Vastag, Gábor Rákhely, Mária A. Deli

Table S1. List of inventoried TaqMan Gene Expression Assays used for quantification of gene expression from rat, human and dog samples. NA: assay not available

**Protein**		**TaqMan Gene Expression Assay**		
		human	***rat***	***canine***
TJ proteins				
OCLN		Hs00170162_m1	Rn00580064_m1	Cf02624089_m1
JAM		Hs00375889_m1	NA	NA
ESAM		Hs00332781_m1	Rn01531509_g1	Cf02650952_m1
CLDN1		Hs01076359_m1	Rn00581740_m1	Cf02713195_u1
CLDN2		Hs00252666_s1	Rn02063575_s1	Cf02624295_g1
CLDN3		Hs00265816_s1	Rn00581751_s1	Cf02624305_s1
CLDN4		Hs00533616_s1	Rn01196224_s1	Cf02695489_s1
CLDN5		Hs01561351_m1	Rn01753146_s1	Cf02701454_u1
CLDN6		Hs00607528_s1	NA	NA
CLDN7		Hs00600772_m1	Rn01496517_g1	Cf02652244_m1
CLDN8		Hs00273282_s1	Rn01767199_s1	NA
CLDN9		Hs00253134_s1	Rn01460292_s1	NA
CLDN10		Hs01075312_m1	NA	Cf02633347_m1
CLDN11		Hs00912957_m1	Rn01293260_m1	Cf02651311_m1
CLDN12		Hs01082669_m1	NA	Cf02707223_u1
CLDN14		Hs00273267_s1	Rn01407193_m1	NA
CLDN15		Hs00204982_m1	Rn02108734_s1	Cf02727618_g1
CLDN16		Hs00198134_m1	Rn00590884_m1	Cf02706284_m1
CLDN17		Hs00273276_s1	NA	NA
CLDN18		Hs00212584_m1	Rn01447445_m1	Cf02710485_g1
CLDN19		Hs00381204_m1	Rn01416539_m1	Cf02728361_m1
CLDN20		Hs00378662_m1	NA	NA
CLDN23		Hs01013638_s1	NA	NA
Influx transporters				
GLUT1	SLC2A1	Hs00892681_m1	Rn01417099_m1	Cf02649247_g1
GLUT3	SLC2A3	Hs00359840_m1	Rn00567331_m1	Cf02627942_m1
GLUT5	SLC2A5	Hs00161720_m1	Rn00582000_m1	Cf02653796_m1
SGLT2	SLC5A2	Hs00894642_m1	Rn00574917_m1	Cf02626898_g1
MCT1	SLC16A1	Hs00161826_m1	Rn00562332_m1	Cf02629517_m1
MCT8	SLC16A2	Hs00185140_m1	Rn00596041_m1	NA

MCT6	SLC16A6	Hs00190779_m1	Rn01460167_m1	NA
CAT1	SLC7A1	Hs00931450_m1	Rn00565399_m1	Cf02741641_m1
LAT1	SLC7A5	Hs00185826_m1	Rn00569313_m1	Cf02671938_m1
XCT	SLC7A11	Hs00204928_m1	Rn01495123_m1	Cf02708143_m1
SAT1	SLC38A1	Hs01562168_m1	Rn00593696_m1	Cf02725832_m1
SAT2	SLC38A2	Hs01089954_m1	Rn00710421_m1	Cf02650100_m1
SN1	SLC38A3	Hs00199177_m1	Rn00595678_m1	NA
SNAT5	SLC38A5	Hs01012028_m1	Rn00593859_m1	Cf02726895_g1
PEPT1	SLC15A1	Hs00953898_m1	Rn00589098_m1	Cf02671905_m1
PEPT2	SLC5A2	Hs00894642_m1	Rn00574917_m1	NA
PHT2	SLC15A3	Hs00275455_m1	Rn00595146_m1	NA
FATP1	SLC27A1	Hs01587917_m1	Rn00585821_m1	Cf02658192_m1
FATP5	SLC27A5	Hs00202073_m1	Rn00577177_m1	NA
ABCA2		Hs00242232_m1	Rn00577821_m1	Cf02643319_m1
ABCA8		Hs00200350_m1	Rn01460836_m1	Cf02654119_m1
MFSD2A		Hs00293017_m1	Rn01516997_m1	Cf02726569_m1
GLYT1	SLC6A9	Hs01042524_m1	Rn01416529_m1	Cf02630605_m1
GLYT2	SLC6A5	Hs00188383_m1	Rn01475607_m1	Cf02702673_m1
TAUT	SLC6A6	Hs00161778_m1	Rn00567962_m1	Cf02628190_m1
CRT	SLC6A8	Hs00940515_m1	Rn00506029_m1	Cf02704862_m1
SMVT	SLC5A6	Hs00221573_m1	Rn00590633_m1	Cf02639308_m1
ASCT1	SLC1A4	Hs00161719_m1	Rn01786205_m1	Cf02649495_m1
ASCT2	SLC1A5	Hs00194540_m1	Rn00598400_m1	Cf02630489_m1
DAT	SLC6A3	Hs00997374_m1	Rn00562224_m1	Cf02695127_m1
NET	SLC6A2	Hs00426573_m1	Rn00580207_m1	Cf02695124_m1
SERT	SLC6A4	Hs00169010_m1	Rn00564737_m1	Cf02622512_m1
GAT1	SLC6A1	Hs01104475_m1	Rn00577652_m1	Cf01104477_m1
GAT2	SLC6A13	Hs00213290_m1	Rn00592456_m1	NA
GAT3	SLC6A11	Hs00204459_m1	Rn00577664_m1	Cf02685557_g1
OATP1A2	SLCO1A2	NA	Rn00756233_m1	NA
OATP1B1	SLCO1B1	Hs00272374_m1	NA	NA
OATP1C1	SLCO1C1	Hs00213714_m1	Rn00584891_m1	Cf00908739_m1
Efflux transporters				
Pgp	ABCB1A	Hs00184500_m1	Rn01639253_m1	Cf02622140_m1
Pgp	ABCB1B	NA	Rn00561753_m1	NA
BCRP	ABCG2	Hs01053790_m1	Rn00710585_m1	Cf02627543_m1
MRP1	ABCC1	Hs00219905_m1	Rn00574093_m1	Cf02703882_m1
MRP2	ABCC2	Hs00166123_m1	Rn00563231_m1	Cf02690806_m1
MRP3	ABCC3	Hs00978473_m1	Rn01452854_m1	Cf02702230_m1
MRP4	ABCC4	Hs00988717_m1	Rn01465702_m1	Cf04245100_m1
MRP5	ABCC5	Hs00981087_m1	Rn00588341_m1	Cf02680658_m1
MRP6	ABCC6	Hs01081201_m1	Rn00578778_m1	Cf02741623_m1
MRP8	ABCC8	Hs01093761_m1	Rn00564778_m1	Cf02690717_m1
MRP11	ABCC11	Hs00261567_m1	NA	Cf02632091_m1
MRP9	ABCC12	Hs00264354_m1	Rn01640157_gH	Cf02716365_m1

EAAT1	SLC1A3	Hs00188193_m1	Rn00570130_m1	Cf02623162_m1
EAAT2	SLC1A2	Hs01102423_m1	Rn00568080_m1	Cf02623760_m1
EAAT3	SLC1A1	Hs00188172_m1	Rn00564705_m1	Cf02623755_m1
EAAT4	SLC1A6	Hs00192604_m1	Rn00583283_m1	Cf03022935_m1
EAAT5	SLC1A7	Hs00198515_m1	NA	NA
Metabolic Phase I and II enzymes				
CYP1A1		Hs01054797_g1	Rn00487218_m1	NA
CYP1A2		Hs01070374_m1	Rn00561082_m1	Cf02622292_m1
CYP2B1		NA	Rn01457875_m1	NA
CYP2B6		Hs03044634_m1	NA	NA
CYP2C9		Hs00426397_m1	NA	NA
CYP2C11		NA	Rn00569868_m1	NA
CYP2C21		NA	NA	Cf02623102_m1
CYP2D4		NA	Rn01504629_m1	NA
CYP2D6		Hs02576168_g1	NA	NA
CYP2D15		NA	NA	Cf02621842_m1
CYP2E1		Hs00559367_m1	Rn01759587_m1	Cf02622921_m1
CYP2J2		Hs00951113_m1	NA	NA
CYP2J4		NA	Rn00576482_m1	NA
CYP2R1		Hs01379776_m1	Rn01754616_m1	NA
CYP2S1		Hs00258076_m1	Rn01475871_m1	NA
CYP2U1		Hs00766273_m1	Rn01522408_m1	NA
CYP3A4		Hs00604506_m1	NA	NA
CYP3A62		NA	Rn01409583_m1	NA
CYP3A23		NA	Rn01412959_g1	NA
CYP7A1		Hs00167982_m1	Rn00564065_m1	NA
CYP27A1		Hs01026016_m1	Rn00710297_m1	NA
GSTP1		Hs00943351_g1	Rn00561378_gH	NA
GSTA1		Hs00275575_m1	Rn01757146_m1	NA
GSTA2		NA	Rn04222952_m1	NA
GSTA3		NA	Rn00580416_m1	NA
SULT1A1		Hs00738644_m1	Rn01510633_m1	Cf02625653_m1
SULT1A2		Hs00236895_m1	NA	NA
SULT1C3		NA	Rn00581955_m1	NA
UGT1A1		Hs00153559_m1	Rn00754947_m1	NA
GGT1		NA	Rn00587709_m1	Cf02652050_m1
GGT5		NA	Rn00570305_m1	Cf02651479_m1
GGT7		NA	Rn00522627_m1	Cf02716505_m1
NOS1		NA	Rn00583793_m1	Cf02623191_m1
NOS2		NA	Rn00561646_m1	Cf02622928_m1
NOS3		NA	Rn02132634_s1	Cf02623179_m1
PRKCD		NA	NA	Cf02626956_m1
PRKCG		NA	Rn00440861_m1	NA
PRKCI		NA	NA	Cf02636104_m1
PRKACA		NA	Rn01432302_m1	NA

PRKAR1A	NA	Rn00566036_m1	NA
PRKAR2A	NA	Rn00709403_m1	NA
XDH	NA	Rn00567654_m1	NA
CAT	NA	Rn00560930_m1	NA
SOD1	NA	Rn01477289_m1	NA
GTPBP10	NA	Rn01755457_m1	NA

Table S2. Relative expression of selected genes in rat BBB model (EPA) and epithelial models by TaqMan Gene Expression Assays. (mean ± SD). NA: assay not available

Protein		Gene expression				
		**EPA**	**Caco-2**	**VB-Caco-2**	**MDCK**	**MDCK-MDR1**
OCLN		15268 ± 12425	19271 ± 4770	23762 ± 10841	2452 ± 281	1930 ± 314
JAM		NA	14963 ± 4474	17047 ± 9612	NA	NA
ESAM		62132 ± 51840	157 ± 42	86 ± 48	4 ± 2	6 ± 2
CLDN1		242 ± 178	22588 ± 7865	12869 ± 6032	19063 ± 3142	15187 ± 2219
CLDN2		10 ± 17	724 ± 183	833 ± 556	76638 ± 17595	37481 ± 4353
CLDN3		37 ± 62	4454 ± 1257	2499 ± 941	4464 ± 234	3964 ± 740
CLDN4		90 ± 80	13480 ± 3227	8361 ± 3950	5054 ± 553	5193 ± 346
CLDN5		26072 ± 38802	0 ± 0	0 ± 0	25 ± 5	7 ± 2
CLDN6		NA	127 ± 9	15 ± 5	NA	NA
CLDN7		0 ± 0	3472 ± 629	4458 ± 1574	6522 ± 364	4974 ± 516
CLDN8		0 ± 0	6 ± 5	2 ± 3	NA	NA
CLDN9		23 ± 17	69 ± 26	103 ± 27	NA	NA
CLDN10		NA	0 ± 0	1 ± 2	394 ± 104	5 ± 3
CLDN11		3939 ± 6721	8 ± 3	0 ± 0	0 ± 0	0 ± 0
CLDN12		NA	1803 ± 435	1949 ± 882	2293 ± 324	2805 ± 267
CLDN14		0 ± 0	13 ± 5	10 ± 6	NA	NA
CLDN15		1836 ± 1685	1025 ± 269	452 ± 155	76 ± 14	110 ± 10
CLDN16		0 ± 0	741 ± 93	405 ± 137	5057 ± 1245	4478 ± 342
CLDN17		NA	0 ± 0	0 ± 0	NA	NA
CLDN18		0 ± 0	1 ± 1	0 ± 0	0 ± 0	0 ± 0
CLDN19		155 ± 172	1 ± 1	0 ± 0	16 ± 11	18 ± 4
CLDN20		NA	2 ± 4	1 ± 1	NA	NA
CLDN23		NA	649 ± 150	191 ± 40	NA	NA
GLUT1	SLC2A1	45154 ± 31280	8235 ± 2525	5783 ± 2820	6776 ± 733	8821 ± 609
GLUT3	SLC2A3	17401 ± 12394	23931 ± 5158	17506 ± 5006	12 ± 7	23 ± 5
GLUT5	SLC2A5	4 ± 7	58821 ± 12820	52008 ± 23787	1 ± 2	0 ± 0
SGLT2	SLC5A2	0 ± 0	350 ± 101	381 ± 131	8 ± 7	1 ± 1
MCT1	SLC16A1	9678 ± 5529	2774 ± 786	2214 ± 1113	0 ± 0	0 ± 0
MCT6	SLC16A6	1471 ± 911	13 ± 4	2 ± 2	NA	NA
MCT8	SLC16A2	4445 ± 3067	131 ± 116	0 ± 0	NA	NA
CAT1	SLC7A1	8124 ± 6268	707 ± 213	414 ± 135	2582 ± 394	2493 ± 200
LAT1	SLC7A5	10865 ± 7667	1570 ± 326	644 ± 100	9861 ± 1691	7691 ± 821
XCT	SLC7A11	702 ± 16	282 ± 95	221 ± 102	1278 ± 401	1157 ± 81
SAT1	SLC38A1	1447 ± 1223	4199 ± 1056	2994 ± 1230	4234 ± 344	3881 ± 704
SAT2	SLC38A2	117646 ± 64399	5781 ± 1589	6224 ± 3329	4702 ± 181	3875 ± 260
SN1	SLC38A3	14870 ± 18254	3697 ± 822	2451 ± 945	NA	NA
SNAT5	SLC38A5	66 ± 49	26 ± 5	129 ± 50	937 ± 162	554 ± 28
PEPT1	SLC15A1	0 ± 0	12103 ± 1297	17820 ± 6655	3 ± 3	1 ± 2
PEPT2	SLC5A2	0 ± 0	350 ± 101	381 ± 131	NA	NA
PHT2	SLC15A3	1424 ± 1000	3 ± 3	6 ± 1	NA	NA
FATP1	SLC27A1	2581 ± 1789	326 ± 69	338 ± 108	603 ± 44	634 ± 83
FATP5	SLC27A5	7 ± 6	8 ± 6	20 ± 5	NA	NA
ABCA2		1514 ± 1500	1008 ± 269	1079 ± 332	336 ± 108	295 ± 33
ABCA8		6 ± 9	3 ± 2	5 ± 1	234 ± 45	2 ± 0
MFSD2A		39 ± 32	351 ± 66	213 ± 28	572 ± 149	192 ± 5
GLYT1	SLC6A9	206 ± 294	99 ± 26	174 ± 35	1072 ± 94	946 ± 85
GLYT2	SLC6A5	0 ± 0	0 ± 0	0 ± 0	3 ± 6	0 ± 0
TAUT	SLC6A6	193 ± 283	1581 ± 200	1736 ± 813	6377 ± 579	7592 ± 1286
CRT	SLC6A8	3068 ± 2345	13117 ± 2256	9220 ± 1950	2258 ± 221	2060 ± 294
SMVT	SLC5A6	1659 ± 997	2088 ± 694	2226 ± 964	14397 ± 700	8004 ± 1180
ASCT1	SLC1A4	4014 ± 3195	177 ± 69	24 ± 17	1278 ± 199	1055 ± 77
ASCT2	SLC1A5	5782 ± 3935	6541 ± 1826	4427 ± 2025	8994 ± 742	5904 ± 321
DAT	SLC6A3	0 ± 0	3 ± 3	6 ± 3	0 ± 0	0 ± 0
NET	SLC6A2	187 ± 324	0 ± 0	0 ± 0	1 ± 2	1 ± 2
SERT	SLC6A4	17 ± 30	4497 ± 1404	12183 ± 5310	0 ± 0	1 ± 1
GAT1	SLC6A1	39 ± 68	0 ± 0	0 ± 0	0 ± 0	0 ± 0
GAT2	SLC6A13	8 ± 15	8 ± 2	7 ± 3	NA	NA
GAT3	SLC6A11	4 ± 7	0 ± 0	0 ± 0	0 ± 0	0 ± 0
OATP1A2	SLCO1A2	2377 ± 2455	NA	NA	NA	NA
OATP1B1	SLCO1B1	NA	3 ± 2	0 ± 0	NA	NA
OATP1C1	SLCO1C1	681 ± 707	0 ± 0	1 ± 1	5 ± 1	0 ± 0

Table S3. Relative expression of selected genes in rat BBB model (EPA) and endothelial models by TaqMan Gene Expression Assays. (mean ± SD). NA: assay not available

Protein		Gene expression				
		EPA	GP8	RBE4	D3	D3L
OCLN		15268 ± 12425	234 ± 113	49 ± 10	4281 ± 1036	4040 ± 253
JAM		NA	NA	NA	5360 ± 1054	7472 ± 581
ESAM		62132 ± 51840	4371 ± 1031	6338 ± 4070	1875 ± 1240	1043 ± 80
CLDN1		242 ± 178	4 ± 3	0 ± 0	6679 ± 129	18155 ± 1820
CLDN2		10 ± 17	19 ± 19	7 ± 13	28 ± 19	30 ± 7
CLDN3		37 ± 62	2 ± 1	0 ± 0	96 ± 44	57 ± 11
CLDN4		90 ± 80	20 ± 5	2 ± 1	315 ± 153	269 ± 31
CLDN5		26072 ± 38802	4 ± 1	0 ± 0	18 ± 17	17 ± 12
CLDN6		NA	NA	NA	18 ± 5	601 ± 517
CLDN7		0 ± 0	0 ± 0	0 ± 0	467 ± 57	1461 ± 106
CLDN8		0 ± 0	0 ± 0	0 ± 0	0 ± 0	0 ± 0
CLDN9		23 ± 17	0 ± 0	0 ± 0	111 ± 14	121 ± 29
CLDN10		NA	NA	NA	25 ± 6	28 ± 22
CLDN11		3939 ± 6721	4 ± 4	1 ± 1	33326 ± 2586	37150 ± 2401
CLDN12		NA	NA	NA	3218 ± 116	2881 ± 195
CLDN14		0 ± 0	0 ± 0	0 ± 0	695 ± 90	101 ± 34
CLDN15		1836 ± 1685	830 ± 400	32 ± 6	383 ± 84	381 ± 87
CLDN16		0 ± 0	0 ± 0	0 ± 0	14 ± 4	31 ± 7
CLDN17		NA	NA	NA	0 ± 0	0 ± 0
CLDN18		0 ± 0	11 ± 8	4 ± 2	0 ± 0	0 ± 0
CLDN19		155 ± 172	12 ± 6	2 ± 1	0 ± 0	0 ± 0
CLDN20		NA	NA	NA	0 ± 0	0 ± 0
CLDN23		NA	NA	NA	91 ± 13	157 ± 56
GLUT1	SLC2A1	45154 ± 31280	5404 ± 2382	809 ± 138	9842 ± 3257	11345 ± 2274
GLUT3	SLC2A3	17401 ± 12394	4 ± 2	3852 ± 218	1182 ± 603	1950 ± 139
GLUT5	SLC2A5	4 ± 7	20 ± 11	0 ± 0	2 ± 3	8 ± 2
SGLT2	SLC5A2	0 ± 0	0 ± 0	0 ± 0	0 ± 0	1 ± 2
MCT1	SLC16A1	9678 ± 5529	10280 ± 4386	1060 ± 223	23668 ± 11115	26813 ± 1337
MCT6	SLC16A6	1471 ± 911	378 ± 252	277 ± 70	470 ± 266	657 ± 90
MCT8	SLC16A2	4445 ± 3067	336 ± 134	25 ± 3	11 ± 10	389 ± 111
CAT1	SLC7A1	8124 ± 6268	11374 ± 6909	1512 ± 335	7036 ± 1443	5450 ± 485
LAT1	SLC7A5	10865 ± 7667	17500 ± 9415	5890 ± 537	7286 ± 1482	7525 ± 651
XCT	SLC7A11	702 ± 16	3018 ± 656	1072 ± 83	15258 ± 4267	9744 ± 879
SAT1	SLC38A1	1447 ± 1223	2 ± 3	1188 ± 252	14124 ± 4002	17044 ± 2160
SAT2	SLC38A2	117646 ± 64399	54683 ± 18317	12651 ± 848	55706 ± 16820	50772 ± 9605
SN1	SLC38A3	14870 ± 18254	4 ± 2	5 ± 2	48 ± 15	411 ± 40
SNAT5	SLC38A5	66 ± 49	0 ± 0	0 ± 0	3992 ± 932	6449 ± 508
PEPT1	SLC15A1	0 ± 0	1 ± 3	0 ± 0	2 ± 2	8 ± 7
PEPT2	SLC5A2	0 ± 0	0 ± 0	0 ± 0	0 ± 0	1 ± 2
PHT2	SLC15A3	1424 ± 1000	5 ± 1	11 ± 4	3250 ± 1236	1373 ± 145
FATP1	SLC27A1	2581 ± 1789	1666 ± 801	350 ± 133	694 ± 176	1222 ± 156
FATP5	SLC27A5	7 ± 6	10 ± 9	334 ± 578	12 ± 19	36 ± 18
ABCA2		1514 ± 1500	1435 ± 320	1036 ± 42	622 ± 136	1587 ± 228
ABCA8		6 ± 9	0 ± 0	0 ± 0	2688 ± 1704	1808 ± 63
MFSD2A		39 ± 32	0 ± 0	1 ± 1	338 ± 317	505 ± 129
GLYT1	SLC6A9	206 ± 294	3307 ± 1406	949 ± 145	224 ± 237	827 ± 213
GLYT2	SLC6A5	0 ± 0	1 ± 1	0 ± 0	0 ± 0	1 ± 1
TAUT	SLC6A6	193 ± 283	33 ± 12	57 ± 8	1265 ± 513	2214 ± 404
CRT	SLC6A8	3068 ± 2345	1404 ± 533	499 ± 72	549 ± 220	3639 ± 998
SMVT	SLC5A6	1659 ± 997	1632 ± 633	364 ± 57	1586 ± 425	2254 ± 293
ASCT1	SLC1A4	4014 ± 3195	3533 ± 1389	1731 ± 243	3201 ± 754	4494 ± 396
ASCT2	SLC1A5	5782 ± 3935	59 ± 19	3659 ± 568	5743 ± 1854	8870 ± 1187
DAT	SLC6A3	0 ± 0	0 ± 0	0 ± 0	6 ± 5	63 ± 24
NET	SLC6A2	187 ± 324	19 ± 18	0 ± 0	0 ± 0	1 ± 2
SERT	SLC6A4	17 ± 30	17 ± 13	27 ± 4	14 ± 9	6 ± 2
GAT1	SLC6A1	39 ± 68	0 ± 0	0 ± 0	0 ± 0	11 ± 3
GAT2	SLC6A13	8 ± 15	1 ± 2	0 ± 0	7 ± 8	5 ± 6
GAT3	SLC6A11	4 ± 7	0 ± 0	0 ± 0	0 ± 0	3 ± 3
OATP1A2	SLCO1A2	2377 ± 2455	0 ± 0	0 ± 0	NA	NA
OATP1B1	SLCO1B1	NA	NA	NA	0 ± 0	0 ± 0
OATP1C1	SLCO1C1	681 ± 707	0 ± 0	0 ± 0	0 ± 0	0 ± 0

Supplementary Fig S1. Ratio of the expression of selected genes coding tight junction proteins in a primary rat brain endothelial cell based BBB model (EPA) and in epithelial cell line models (Caco2, VB-Caco2, MDCK and MDCK-MDR1).

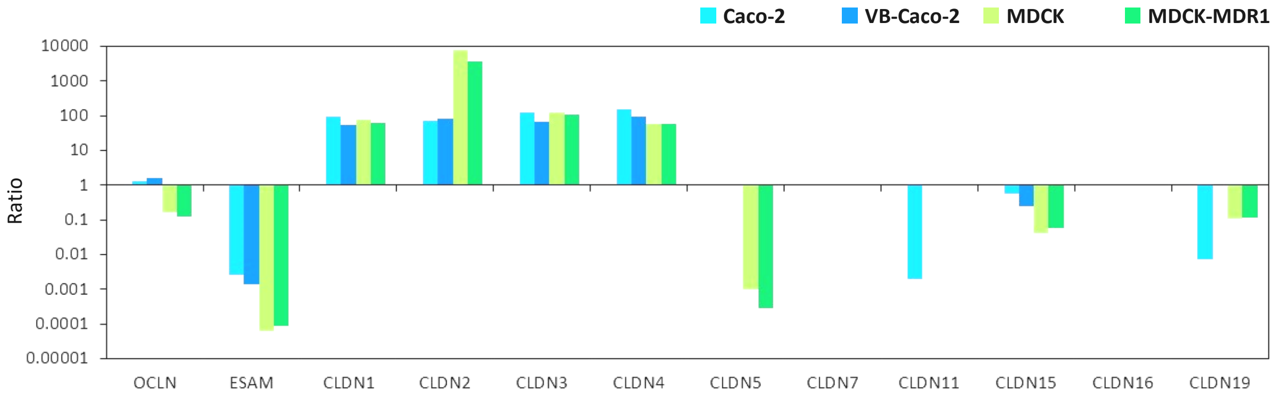

Supplementary Fig S2. Ratio of the expression of selected genes coding solute carriers and other nutrient transporters in a primary rat brain endothelial cell based BBB model (EPA) and in epithelial cell line models (Caco-2, VB-Caco-2, MDCK and MDCK-MDR1). NA: assay not available.

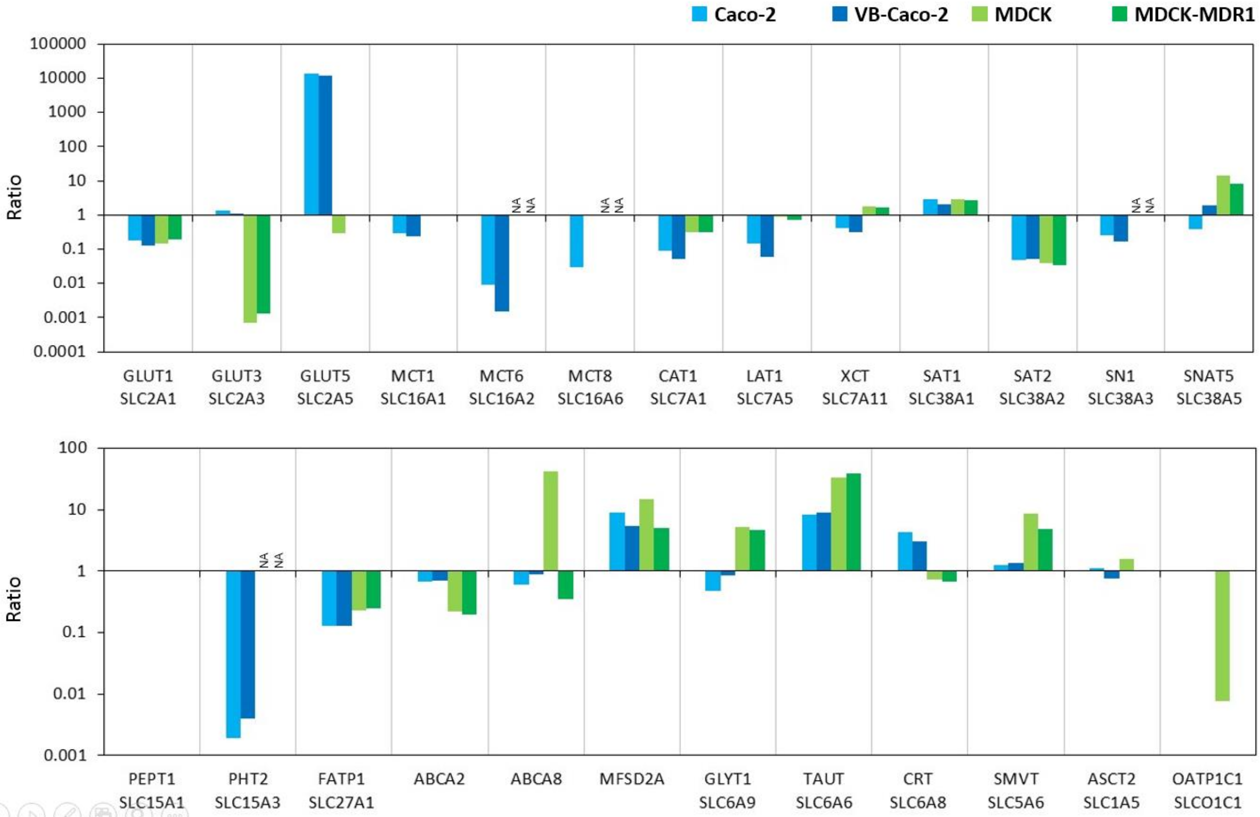

Supplementary Fig S3. Ratio of the expression of selected genes coding efflux transporters in a primary rat brain endothelial cell based BBB model (EPA) and in epithelial cell line models (Caco-2, VB-Caco-2, MDCK and MDCK-MDR1).

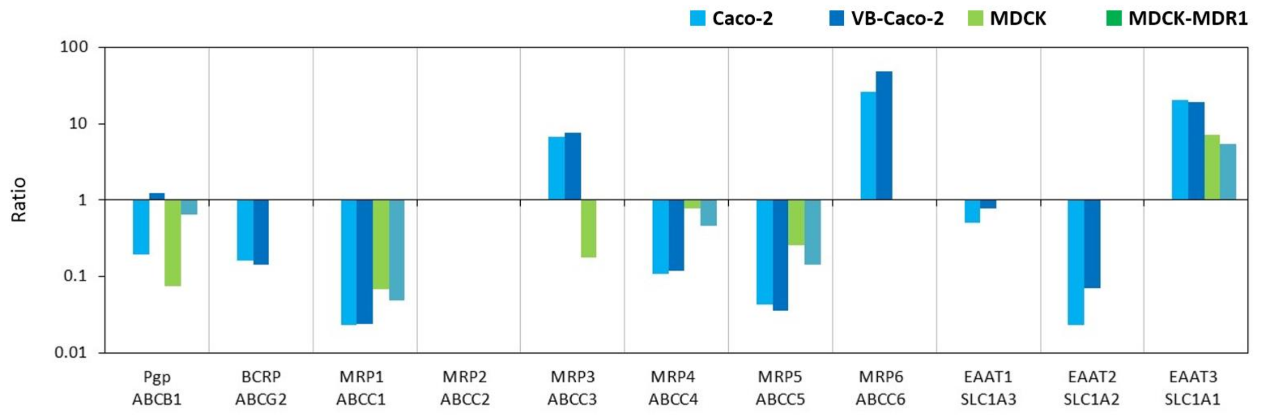

Supplementary Fig S4. Ratio of the expression of selected genes coding metabolic enzymes in a primary rat brain endothelial cell based BBB model (EPA) and in epithelial cell line models (Caco-2, VB-Caco-2, MDCK and MDCK-MDR1). NA: assay not available

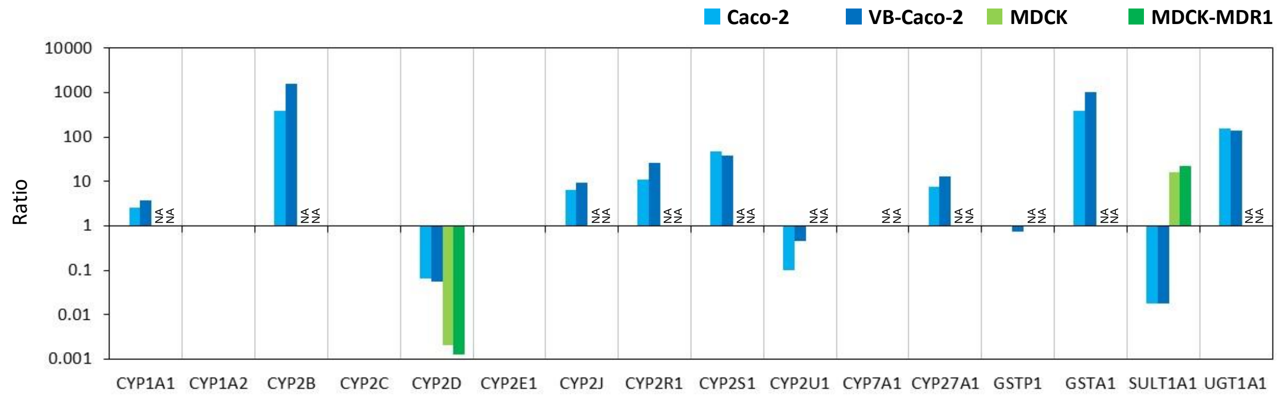

Supplementary Fig S5. Ratio of the expression of selected genes coding tight junction proteins in a primary rat brain endothelial cell based BBB model (EPA) and in brain endothelial cell line models (GP8, RBE4, D3 and D3L).

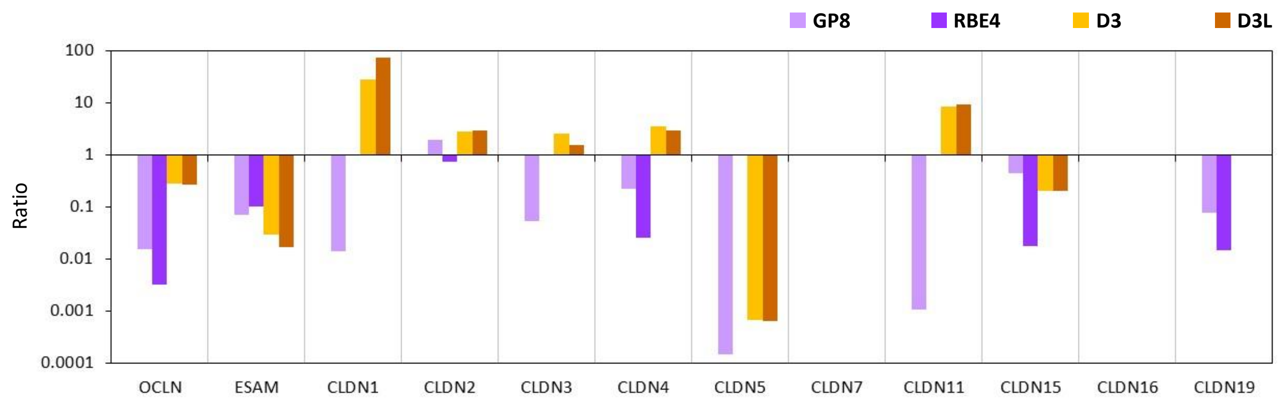

Supplementary Fig S6. Expression of selected genes coding solute carriers and other nutrient transporters in a primary rat brain endothelial cell based BBB model (EPA) and in brain endothelial cell line models (GP8, RBE4, D3 and D3L).

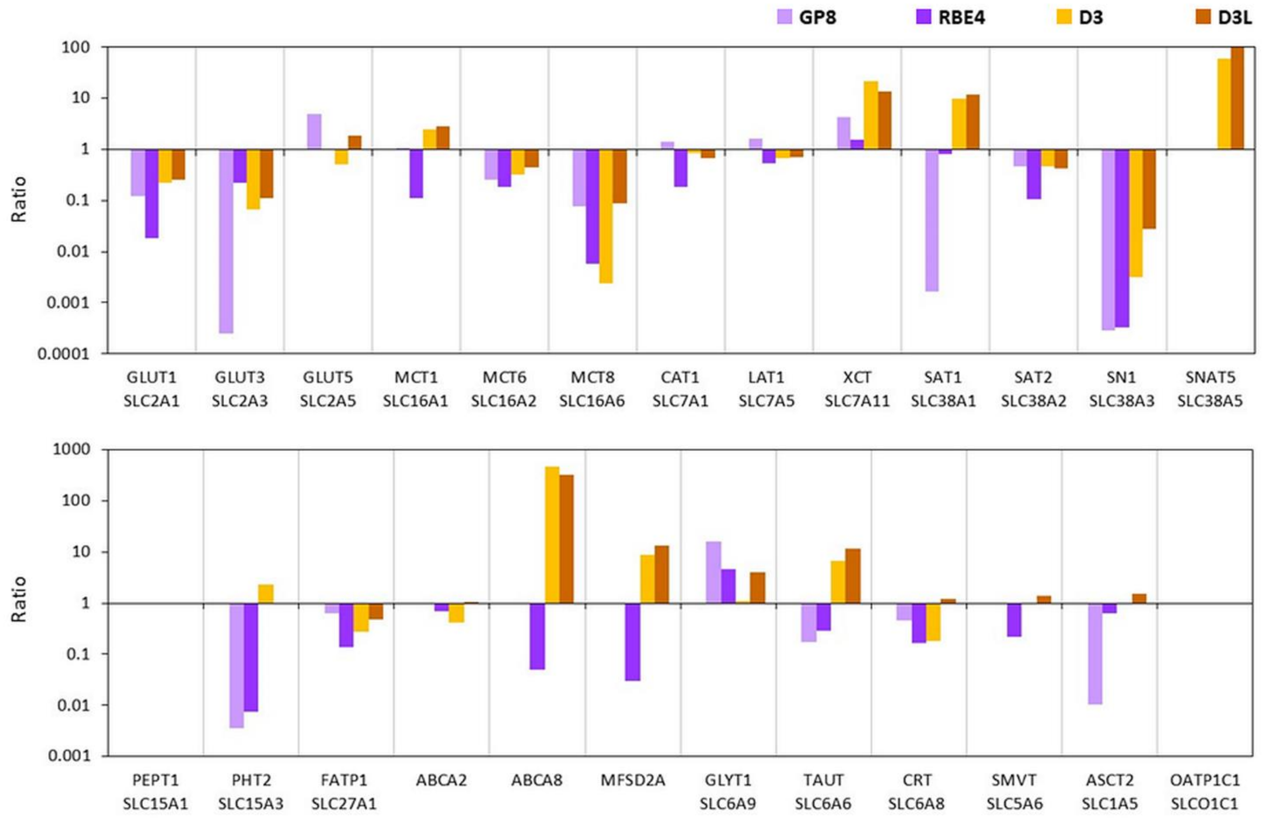

Supplementary Fig S7. Expression of selected genes coding efflux transporters in a primary rat brain endothelial cell based BBB model (EPA) and in brain endothelial cell line models (GP8, RBE4, D3 and D3L).

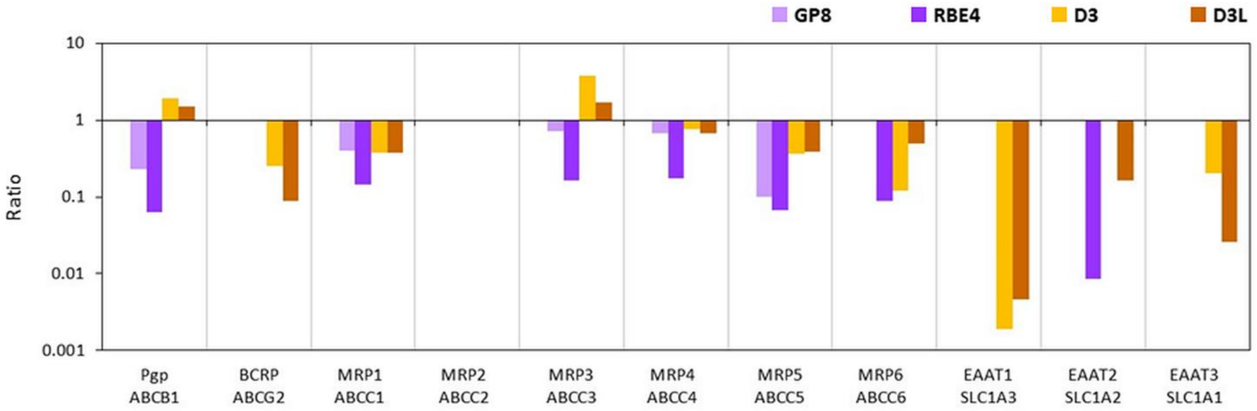

Supplementary Fig S8. Expression of selected genes coding metabolic enzymes in a primary rat brain endothelial cell based BBB model (EPA) and in brain endothelial cell line models (GP8, RBE4, D3 and D3L).

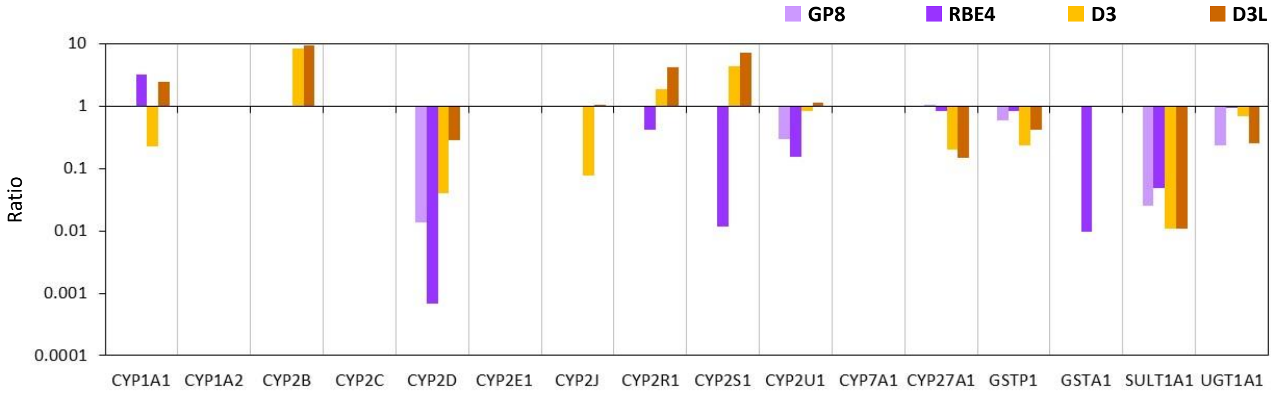

Original, non-merged images for Figure 9.

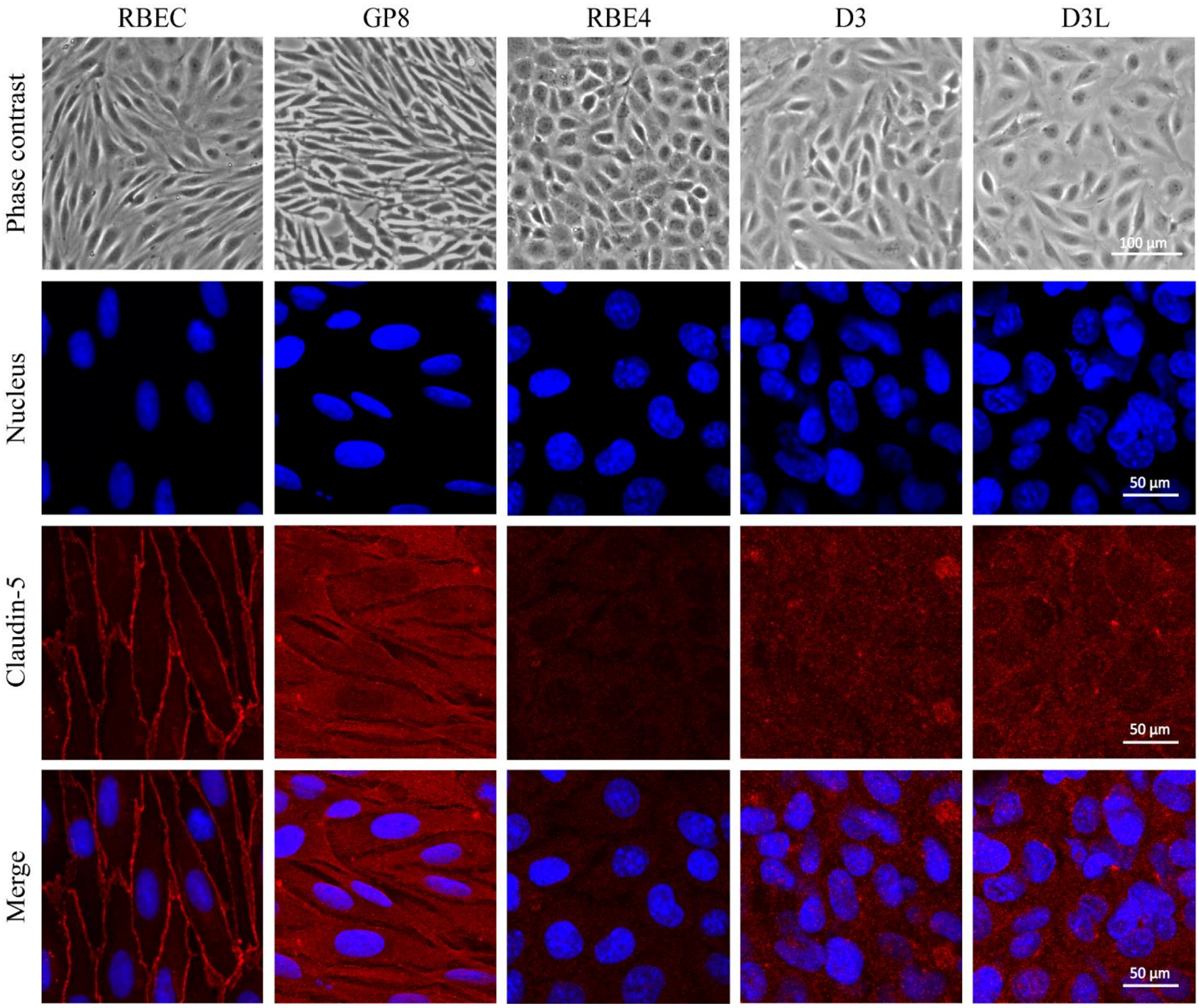

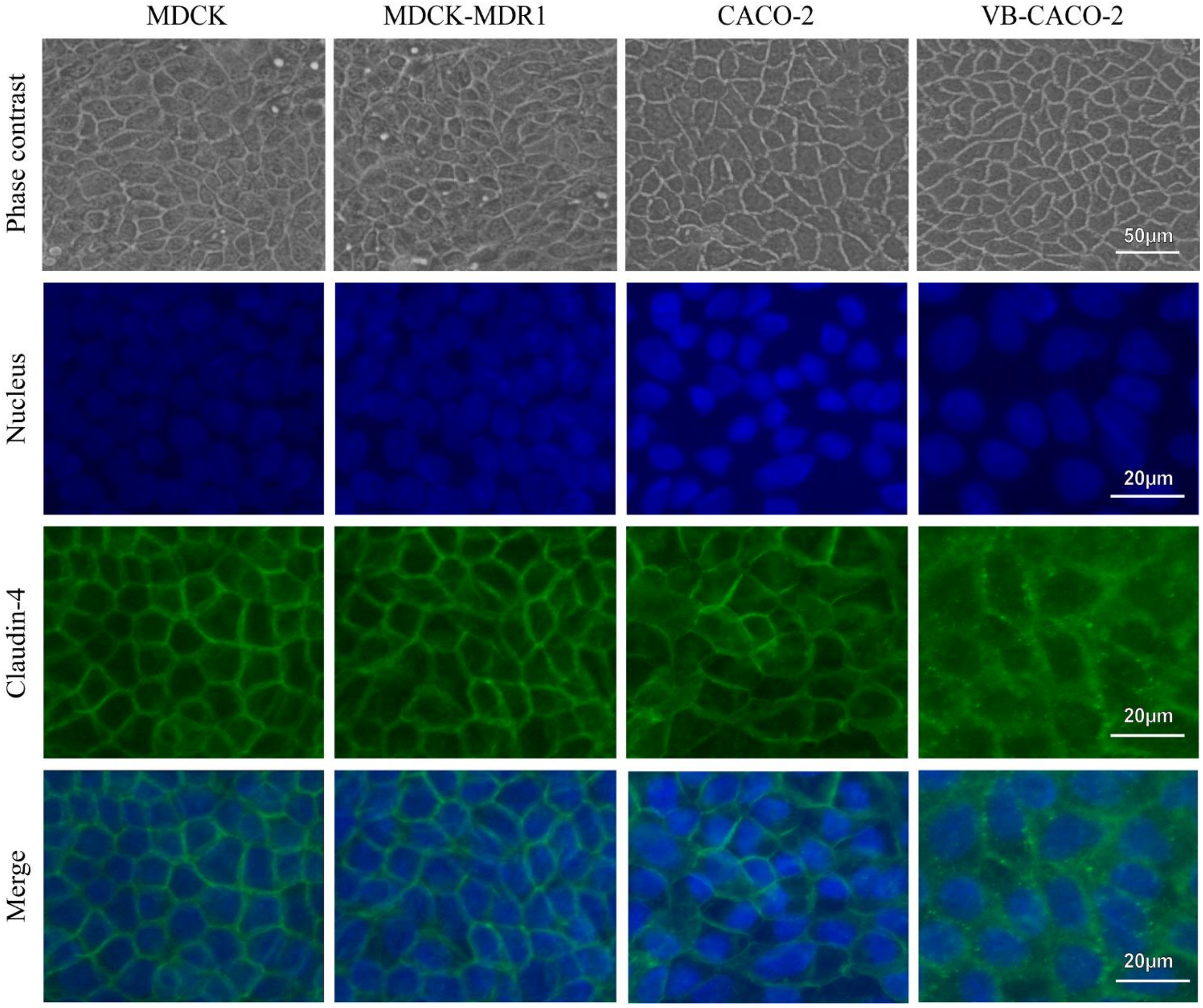
MDCK
MDCK-MDR1
CACO-2
VB-CACO-2
Phase contrast
Nucleus
Claudin-4
Merge
50μm
20μm
20μm
20μm
